# The Recurrent-Specific Regulation Network of Prognostic Stemness-Related Signatures in Low-Grade Glioma

**DOI:** 10.1155/2023/2243928

**Published:** 2023-01-17

**Authors:** Jin Li, Meng Zhou, Dan Huang, Ruoyi Lin, Xiaomei Cui, Shaofeng Chen, Ying Yao, Shuyuan Xian, Siqiao Wang, Qing Fu, Jiwen Zhu, Xi Yue, Runzhi Huang, Enbo Qi, Zongqiang Huang

**Affiliations:** ^1^Department of Orthopedics, The First Affiliated Hospital of Zhengzhou University, Zhengzhou 450052, China; ^2^Department of Comprehensive Chemotherapy, Hunan Cancer Hospital, 283 Tongzipo Road, Changsha, Hunan, China; ^3^Tongji University School of Medicine, Shanghai 200092, China; ^4^Department of Operating Room, Tongji Hospital, School of Medicine, Tongji University, Shanghai 200065, China; ^5^Department of Orthopaedic Surgery, Changhai Hospital, Navy Military Medical University, Shanghai 200433, China; ^6^Division of Spine, Department of Orthopedics, Tongji Hospital Affiliated to Tongji University School of Medicine, Shanghai 200065, China; ^7^Department of Neurosurgery, Xinhua Hospital Affiliated to Shanghai Jiao Tong University School of Medicine, Shanghai 200092, China

## Abstract

Gliomas including astrocytomas, oligodendrogliomas, mixed oligoastrocytic, and mixed glioneuronal tumors are an important group of brain tumors. Based on the 2016 WHO classification for tumors in the central nervous system, gliomas were classified into four grades, from I to IV, and brain lower grade glioma (LGG) consists of grade II and grade III. Patients with LGG may undergo recurrence, which makes clinical treatment tough. Stem cell-like features of cancer cells play a key role in tumor's biological behaviors, including tumorigenesis, development, and clinical prognosis. In this article, we quantified the stemness feature of cancer cells using the mRNA stemness index (mRNAsi) and identified stemness-related key genes based on correlation with mRNAsi. Besides, hallmark gene sets and translate factors (TFs) which were highly related to stemness-related key genes were identified. Therefore, a recurrency-specific network was constructed and a potential regulation pathway was identified. Several online databases, assay for transposase-accessible chromatin using sequencing (ATAC-seq), single-cell sequencing analysis, and immunohistochemistry were utilized to validate the scientific hypothesis. Finally, we proposed that aurora kinase A (AURKA), positively regulated by Non-SMC Condensin I Complex Subunit G (NCAPG), promoted E2F target pathway in LGG, which played an important role in LGG recurrence.

## 1. Introduction

Gliomas are an important group of brain tumors, which include astrocytomas, oligodendrogliomas, mixed oligoastrocytic, and mixed glioneuronal tumors. They often originate from supporting glial or precursor cells in the brain [1, 2]. Gliomas account for 25.5% of all central nervous system (CNS) tumors, and 80.8% of these are malignant [3]. According to the 2016 WHO classification of tumors of the CNS, gliomas were classified into several different types and four grades from grade I to grade IV based on their phenotype and genotype. Brain lower grade glioma (LGG) consists of grade II and grade III, commonly occurring in people from 30 to 40 years old, with various symptoms and mass effects like invasions, compression, or obstructions [4, 5]. Generally, patients with LGG have various clinical outcomes: some patients follow a longer lifetime, while other patients undergo recurrence, deterioration, and malignant transformation, which make the therapy tough [6]. The overall survival rates at 5, 10, and 15 years of patients with LGG were 38%, 18%, and 1%, respectively, and recurrence rates of grade I and grade II were 84.5% and 57.6% [7]. Previous research has shown that several genes were altered in LGG, which played important roles in clinical progress and prognosis. The alteration of IDH1/2 and chromosomes 1 and 19 has been wildly proved to have close relationship with increased survival and recurrent rate [8, 9]. However, the present information is far from enough to understand the genetic regulation mechanism of biological characteristics like recurrence and how that affects clinical prognosis. Therefore, more researches still need to be done.

In silico techniques have been widely used in precision medicine researches; it utilizes high-throughput screening techniques based on genomics, transcriptomics, proteomics, metabolomics, or multiomics to explore the biological mechanism and potential treatment of diseases [10]. Rajendran et al. have applied in silico techniques to the diagnosis and treatment of various diseases, such as tuberculosis, dengue, and cancer [11–14]. Besides, computational single-cell RNA sequencing (scRNA-seq) analysis was very popular in studying the cellular composition and heterogeneity within the tumors based on transcriptomics [15–17]. Assay for transposase-accessible chromatin with high-throughput sequencing (ATAC-seq) and chromatin immunoprecipitation sequence (ChIP-seq) are valuable tools to study the relationship between translational factors and gene expression [18, 19]. What is more, in silico models have been widely used to investigate biological events within tumor tissues [20, 21].

It is widely accepted that cancer cells possess stem cell-like features including loss of the differentiated phenotype and the capacity to self-renew, which play a key role in tumorigenesis, development, and clinical prognosis [22]. The stemness feature of cancer cells could be quantified using the mRNA stemness index (mRNAsi) at the gene expression level [23]. In this article, the differential expression genes (DEGs) between primary and recurrent LGGs were identified using edgeR, and the mRNAsi of every DEGs was calculated by machine-learning algorithm. Then, using weighted correlation network analysis (WGCNA), DEGs were classified into several modules and genes in the same module were strongly correlated. Phenotypic characteristics of each module were annotated through coanalysis with hallmark gene sets, mRNAsi, and modules. Genes with high correlation with mRNAsi were defined as stemness-related key genes, and hallmark gene sets remarkably correlated with modules were defined as key hallmark gene sets. Then, the univariate and multivariate Cox proportional hazard regression was conducted to select prognostic genes. To illuminate the contribution of immunity in LGG recurrence, CIBERSORT estimation was applied to analyze the infiltrating immune cells in LGG. The coanalysis for prognostic genes, differentially expressed translate factors (TFs), and key hallmark gene sets was conducted using the Pearson correlation analysis. Then, a recurrency-specific network was constructed and a potential regulation pathway was identified. Finally, to validate the scientific hypothesis, single-cell sequencing analysis was conducted to validate the distribution of key gene expression in LGG cells at a cellular level, and immunohistochemistry was conducted to validate the expression level of key TFs and key genes at the histological level. What is more, assay for transposase-accessible chromatin using sequencing (ATAC-seq), chromatin immunoprecipitation sequence (ChIP-seq), and multiple databases were applied.

Puromycin works on inhibiting protein synthesis by interfering the translation process [24]; previous studies have proven its antibiotic properties and its antitumor effects [25, 26]. In this study, Connectivity Map (CMap) analysis was used to screen the specific inhibitors of the potential pathway, and puromycin was proven to be the specific inhibitor working on the potential pathway in LGG, which provided a potential treatment strategy for LGG.

Since the present information is not enough to understand the genetic regulation mechanism and predict prognosis of LGG patients, our study is aimed at constructing the recurrent-specific regulation network of prognostic stemness-related signatures to reveal the underlying stemness-related mechanism and predict prognosis of LGG patients. In our study, we quantified the stemness feature of cancer cells using the mRNA stemness index (mRNAsi) and identified stemness-related key genes. Besides, we constructed a recurrency-specific network and identified a potential regulation pathway to predict the potential biological mechanism in LGG recurrence. In summary, our study may provide biological mechanism and potential therapy target for LGG recurrence.

## 2. Methods

### 2.1. Data Acquisition

The Cancer Genome Atlas (TCGA) database (https://portal.gdc.cancer.gov/) is an authoritative and freely accessible platform that provides comprehensive information about cancer genetics [27, 28]. RNA sequencing data and clinical information of 531 LGG samples were downloaded from TCGA database, including 511 samples of the primary tumor and 20 samples of recurrent tumor. Data of 2,498 immune genes were downloaded from the ImmPort (https://www.immport.org/) and MsigDB (https://software.broadinstitute.org/gsea/msigdb/) database. Data of 318 transcription factors were downloaded from the Cistrome database (http://cistrome.org). 50 hallmark gene sets were downloaded from the Molecular Signatures Database (MSigDB) v7.0 (https://www.gsea-msigdb.org/gsea/msigdb/genesets.jsp?collection=H) [29]. Additionally, to validate the gene expression level on the cellular level, the single-cell sequencing (scRNA-seq) data was downloaded from Gene Expression Omnibus (GEO) (https://www.ncbi.nlm.nih.gov/geo/query/acc.cgi?acc=GSE164041).

### 2.2. Identification of Differentially Expressed Genes

Based on the edgeR package, DEGs, including differentially expressed TFs, between samples of the primary tumor and recurrent tumor were screened out. Two criteria must be fitted at the same time: the absolute value of log_2_ fold change (log_2_FC) > 1, and the false discovery rate (FDR) < 0.05. To clarify the annotations of DEGs, the Gene Ontology (GO) and Kyoto Encyclopedia of Genes and Genomes (KEGG) enrichment analyses were performed.

### 2.3. Acquisition of the mRNA Stemness Index

The mRNA stemness index (mRNAsi) of 536 primary and recurrent LGG samples was obtained by using the one-class logistic regression machine learning algorithm (OCLR) following Malta et al.'s method [23].

### 2.4. Weighted Gene Coexpression Network Analysis

Weighted correlation network analysis (WGCNA) has been widely used to build and summarize modules consisting of high interconnected genes [30]. DEGs were classified into several modules using the WGCNA R package, and a gene coexpression network was constructed using Pearson correlation analysis. Here, a soft threshold was utilized to define the power parameter. The principal component of a specific module represented the gene expression profiling in the module, and module eigengenes (MEs) were defined as the principal component. Module membership (MM) showed the correlation between the gene expression profile of a given module and its module eigengenes.

To find the biological phenotype of each module, coanalyses for hallmark gene sets, mRNAsi, and modules were conducted. Gene significance (GS) was a measurement for the correlation between phenotypes and genes, and the higher the absolute value of GS, the greater the significance of the biological correlation between phenotypes and genes. The module significance (MS) was a measurement of the correlation between modules and phenotypes, and it was obtained by calculating the average absolute GS of all genes in a specific module.

Stemness-related key genes were selected from modules for further analysis. The criteria for selecting were as follows: the absolute value of GS between mRNAsi and genes was more than 0.50, and the MM was more than 0.50. Also, according to the GS with key genes, the key hallmark gene sets were selected as the potential pathway for further analysis.

### 2.5. Construction of Multivariate Prognosis Model

Firstly, the univariate Cox proportional hazard regression was used to select prognostic genes, and stemness-related key genes with HR > 1 and *P* value < 0.5 were considered as prognostic genes. The Least Absolute Shrinkage and Selection Operator (LASSO) regression was used to eliminate overfitting. And for each sample with LGG, the risk score was calculated by the following formula:

(1) $$\begin{array}{c} \text{Risk score}=\text{C}1\times \text{DEG}1+\text{C}2\times \text{DEG}2+\text{C}3\times \text{DEG}3\cdots +Cn\times \text{DEG}n. \end{array}$$

For every single sample, “*n*” represented the number of DEG in the multivariate model, “*C*” represented the regression coefficient of each DEG, and DEG*n* represented the expression level of the *N*th DEG in the corresponding sample. According to the median risk score, all patients were classified into two groups: high-risk and low-risk groups. Receiver operating characteristic (ROC) curve was conducted to assess the accuracy. The Kaplan-Meier curve was conducted to predict the prognosis value of the risk score. Besides, univariate Cox proportional hazard regression was performed to validate the prognostic value of the risk score and other factors like age, gender, and race. Multivariate Cox proportional hazard regression was conducted to assess whether the risk score was an independent prognostic factor.

The correlation between the risk groups and clinical characteristics was analyzed, and the *χ*^2^ test was applied to the censor group and immune subtype group. What is more, to further explore the potential downstream pathway of patients in the high-risk group, the Gene Ontology (GO) and Kyoto Encyclopedia of Genes and Genomes (KEGG) enrichment analyses were performed, and by using GSEA, the expression levels of 50 hallmark gene of cancers, KEGG pathway, and GO pathway were identified.

### 2.6. CIBERSORT Estimation to Analyze the Infiltrating Immune Cells

CIBERSORT algorithm was used to identify the infiltrating immune cells and immune function in LGG cells [31]. After the CIBERSORT processing, the inclusion criteria were *P* < 0.05, and the eligible samples were used for further studies. The Wilcoxon rank test was used to identify immune cells, which were distributed and significantly different between patients with high risk and low risk, and the correlation of those different distributed immune cells and the overall survival of patients with LGG were explored using Kaplan-Meier analyses.

### 2.7. Identification of Potential Signal Axis

Using GSVA, the quantification of gene expression in 50 hallmark gene sets of each sample was calculated. Then, differential expression analysis between primary and recurrent patients was conducted. Based on these 50 hallmark gene sets, Gene Set Enrichment Analysis (GSEA) was applied to investigate the expression level of each hallmark gene set in primary and recurrent samples [32], and upregulated hallmark gene sets in recurrent samples were obtained. Finally, the intersection of differential expressed hallmark gene sets in GSVA, GSEA, and module phenotypic traits was defined as the key pathway for the following analysis.

The Pearson correlation analysis was applied between key pathways, TFs, and prognostic genes. Eventually, the regulatory network among TFs, prognostic genes, and key pathways was constructed. What is more, a protein-protein interaction network (PPI) was obtained by using the STRING database [33].

### 2.8. Connectivity Map Analysis

To clarify the systematic connection between potential pathways and drug actions, the Connectivity Map (build 02) (https://portals.broadinstitute.org/cmap/) was used to screen out the inhibitors of the potential pathway [34]. Differentially expressed mRNAs (DEmRNAs) identified in our study using WGCNA and DEGs in 33 TCGA pancancer were input as queries into CMap. CMap instance was measured by an enrichment score, which ranged from -1 to 1. When enrichment score was more close to -1, those queries were anticorrelated to the drug action, which means the drug could be considered as a promising therapeutic agent that acts as a specific inhibitor of the key pathway [35, 36].

### 2.9. ATAC-seq and ChIP-seq Validation

ATAC-seq is a powerful approach to clarify genome-wide chromatin accessibility, based on the use of Tn5 transposase with adaptors to fragment open chromatin and tag sequencing adaptors in genome [37]. In this study, ATAC-seq data of LGG samples were downloaded from the chromatin accessibility landscape of primary human cancers in TCGA database (https://gdc.cancer.gov/about-data/publications/ATACSeq-AWG) to explore the accessible chromatin of LGG cells and the specific interactive location of key TFs and prognostic key genes [18], and the binding relationship between the key TFs and prognostic key genes was performed using Gviz package [38, 39]. What is more, chromatin immunoprecipitation sequencing (ChIP-seq) data was downloaded from the Cistrome database to validate the directly binding relationship between key TFs and key genes [19].

### 2.10. Single-Cell RNA Sequencing Analysis

To validate the expression level and distribution of the key genes in LGG cells on cellular level, the single-cell sequencing (scRNA-seq) data was downloaded from Gene Expression Omnibus (GEO) https://www.ncbi.nlm.nih.gov/geo/query/acc.cgi?acc=GSE164041). We used the Seurat pipeline to analyze those data [15]. After normalizing the data by removing cells that did not meet the criteria and identifying variable genes using “vst” method, principal component analysis (PCA) was applied to filter genes based on the expression level of variable genes, and top 15 PCs were selected and the Uniform Manifold Approximation and Projection (UMAP) method was utilized to reduce dimension and identify different cell clusters. Furthermore, each cluster was annotated using the SingleR method [40] and CellMarker database [41].

### 2.11. Immunohistochemistry Validation

Paraffin-embedded and fixed tissues of diagnostic biopsies from LGG patients (primary patients and recurrent patients) were collected and were incubated with antibodies overnight at 4°C, the antibodies came from Abcam, and the contribution was 1 : 300. Then, after three times washing with PBS, those tissues were incubated with secondary antibody for 1 hour. The slides were stained with 3,3-diaminobenzidine tetrahydrochloride (DAB), and the nuclei were counterstained with haematoxylin. Immunostaining level was accessed in each slide to detect the expression level of AURKA and NCAPG in tumor cells between primary patients and recurrent patients. Besides, immunohistochemistry staining data of brain normal tissues and gliomas tissues were downloaded from the Human Protein Atlas (https://www.proteinatlas.org).

### 2.12. Multidimensional Validation

To further validate the hypothetical signal pathway from several aspects, the top five genes from the key pathways were selected by GeneCards (https://www.genecards.org/). Then, correlations between LGG and those genes in the scientific hypothesis were further validated by multiple online databases including Gene Expression Profiling Interactive Analysis (GEPIA) [42], Oncomine [43], cBioPortal [44], UALCAN [45], LinkedOmics [46], and TISIDB [47].

## 3. Results

### 3.1. Identification of Differentially Expressed Genes

**Figure 1:**
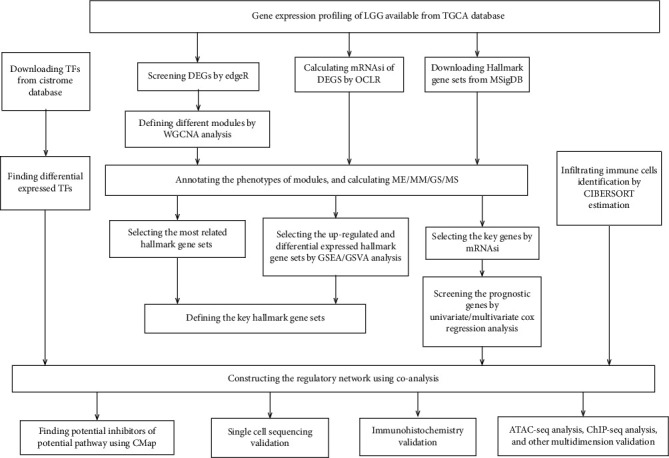
Work flowchart of the study.

The RNA sequencing of 536 LGG patients was obtained from TCGA database. All patients' clinical information was summarized in Table 1. The analysis process is summarized in Figure 1.

**Figure 2:**
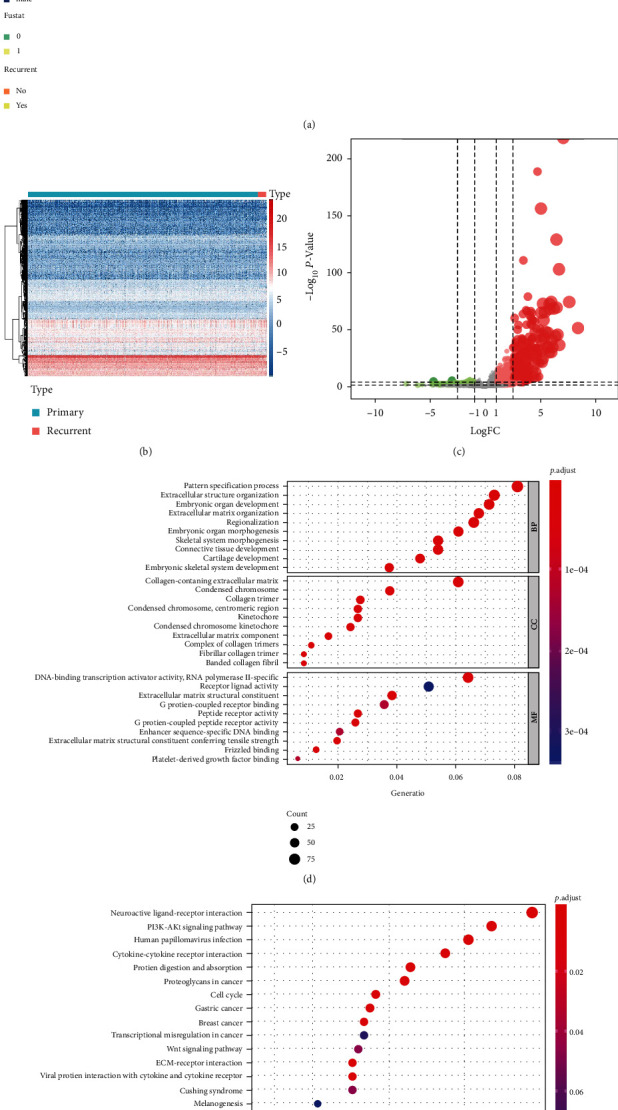
Differentially expressed genes (DEGs) in primary and recurrent low-grade glioma (LGG): (a) mRNA stemness index (mRNAsi) of DEGs in primary and recurrent LGG samples and their demographics data; (b) the heat map of gene expression level in primary and recurrent gliomas; (c) the volcano plots showed DEGs in primary versus recurrent samples; (d, e) the GO and KEGG enrichment analysis for DEGs to clarify the annotation; BP means biological process, CC means cellular component, and MF means molecular function.

DEGs were screened out by using edgeR, and 2,147 DEGs were found. The mRNAsi (Figure 2(a)), the heat map of gene expression information (Figure 2(b)), and the volcano plots of DEGs (Figure 2(c)) were presented. What is more, the GO (Figure 2(d)) and KEGG (Figure 2(e)) enrichment analyses for DEGs were conducted. The results showed that pattern specification process (BP, gene ratio = 0.081, *P* < 0.001, count = 93), collagen-containing extracellular matrix (CC, gene ratio = 0.061, *P* < 0.001, count = 73), and RNA polymerase II-specific (MF, gene ratio = 0.064, *P* < 0.001, count = 72) were the most remarkable GO items, and neuroactive ligand-receptor interaction (gene ratio = 0.097, *P* < 0.001, count = 51) was the most remarkable KEGG item.

### 3.2. WGCNA

**Figure 3:**
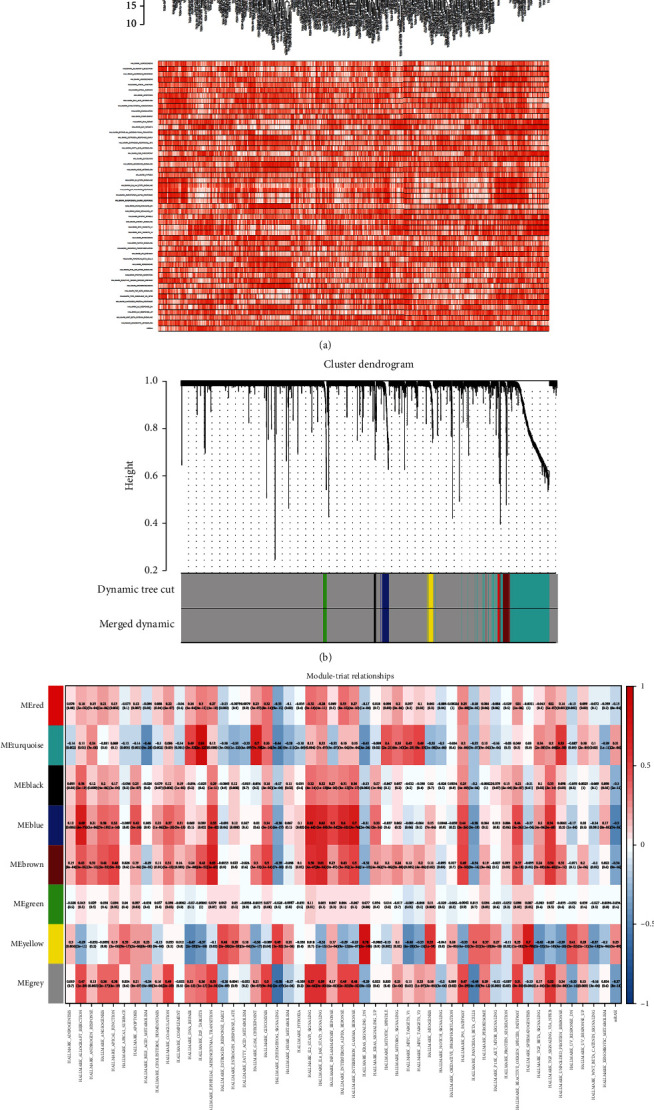
Weighted correlation network analysis (WGCNA) of DEGs: (a, b) the DEGs were divided into eight modules based on WGCNA; each colors represented a module; (c) co-expression analysis for 50 hallmark gene sets, mRNAsi, and modules.

DEGs were divided into eight modules based on WGCNA package (Figures 3(a) and 3(b)). The results of coexpression analysis for 50 hallmark gene sets, mRNAsi, and modules are presented in Figure 3(c). On the basis of correlation with mRNAsi, genes in all modules except green and grey modules were selected as stemness-related key genes for the subsequent analysis. Moreover, six hallmark gene sets which were remarkably correlated with those modules were selected as the key hallmark gene sets, including hallmark E2F targets, hallmark myc targets V2, hallmark G2M checkpoint, hallmark DNA repair, hallmark unfolded protein response, and hallmark spermatogenesis.

### 3.3. Construction of Multivariate Prognostic Model

**Figure 4:**
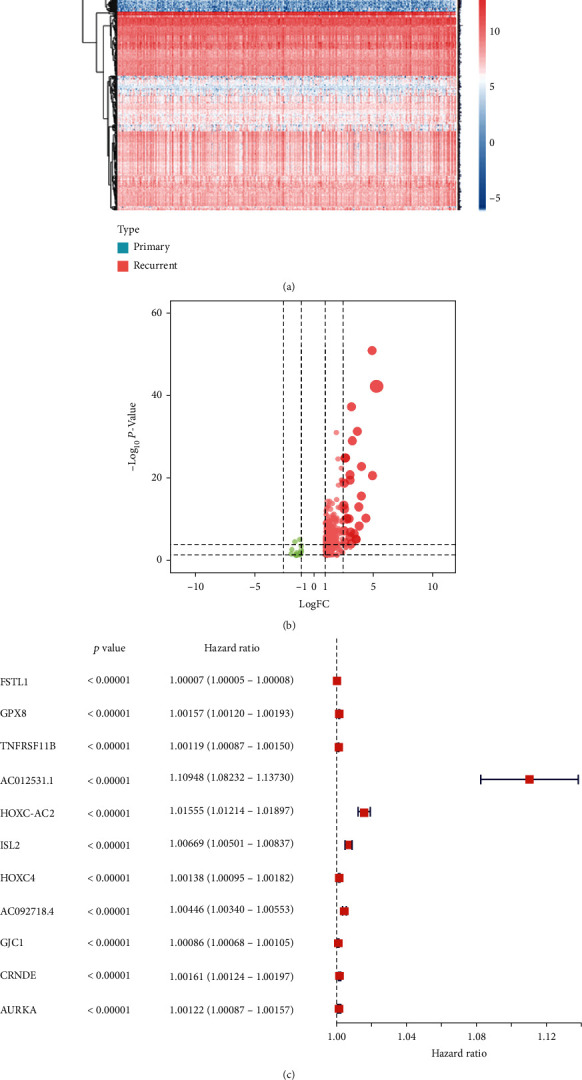
Identification of prognosis-related key genes. (a) The heat map plot showed the expression level of stemness-related genes in primary and recurrent LGG. (b) The volcano plot showed different expressed genes of stemness-related genes. (c) The forest plot showed the results of the univariate Cox regression analysis, and 11 stemness-related genes were identified as prognosis-related key genes.

**Figure 5:**
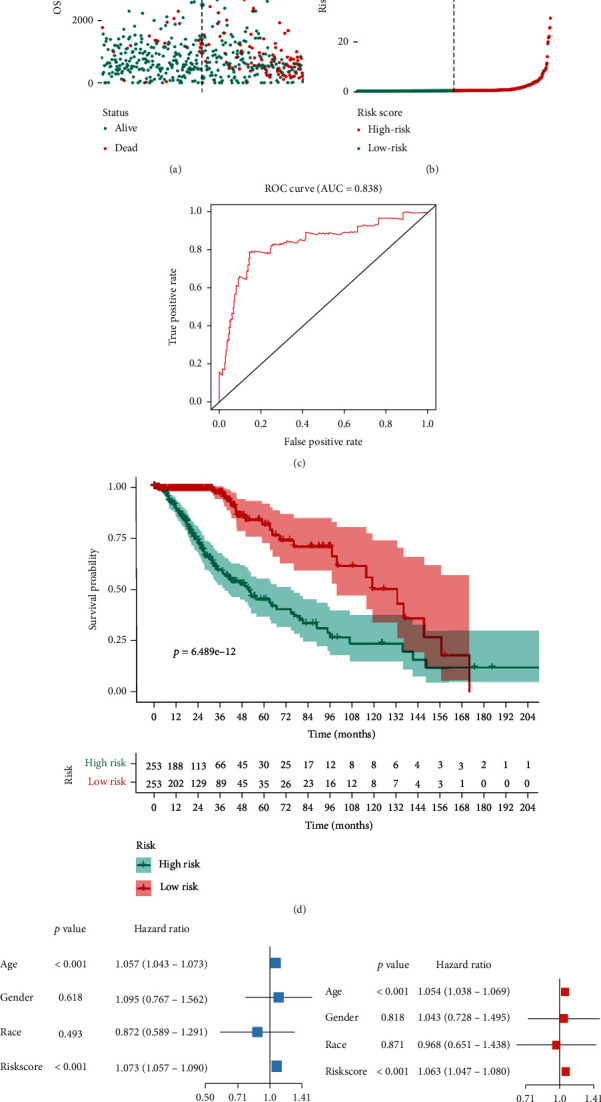
Model validation of the risk score as an independent prognostic factor of LGG recurrency. (a) Risk score of each patients was obtained using multivariate Cox regression analysis for prognosis-related genes. (b) The risk line plot showed the distribution of patients between low- and high-risk groups according to risk score. (c) The area under curve (AUC) of ROC curve was 0.838, indicating a good predict power of the model. (d) Overall survival analysis of patients with LGG between low- and high-risk groups; the high-risk group had a worse clinical outcome. (e) The univariate Cox regression model showed risk score was significantly related prognosis. (f) The multivariate Cox regression model confirmed that the risk score of prognosis-related genes was an independent prognostic factor of LGG recurrency.

The heat map (Figure 4(a)) and volcano (Figure 4(b)) plot of stemness-related key genes were displayed. And the prognostic key genes were selected using univariate Cox regression analysis, and the results were presented in the forest plot (Figure 4(c)). Then, the multivariate Cox regression analysis was conducted for prognostic genes to obtain the risk score of each patient (Figure 5(a)), and the risk line plot (Figure 5(b)) illuminated the distribution of patients in low-risk and high-risk groups.

To assess the accuracy, the area under the curve (AUC) of the ROC curve was 0.984 (Figure 5(c)). The Kaplan-Meier survival analysis (Figure 5(d)) showed a significant difference between low-risk and high-risk groups, and the high-risk group had a worse prognosis. The univariate Cox regression (Figure 5(e)) (HR = 276.97, 95% CI (81.653-939.550), *P* < 0.001) showed a high prognostic value of risk score. Then, the multivariate (Figure 5(f)) (HR = 1.063, 95% CI (1.047-1.080), *P* < 0.001) Cox regression confirmed that the risk score was an independent prognostic factor.

**Figure 6:**
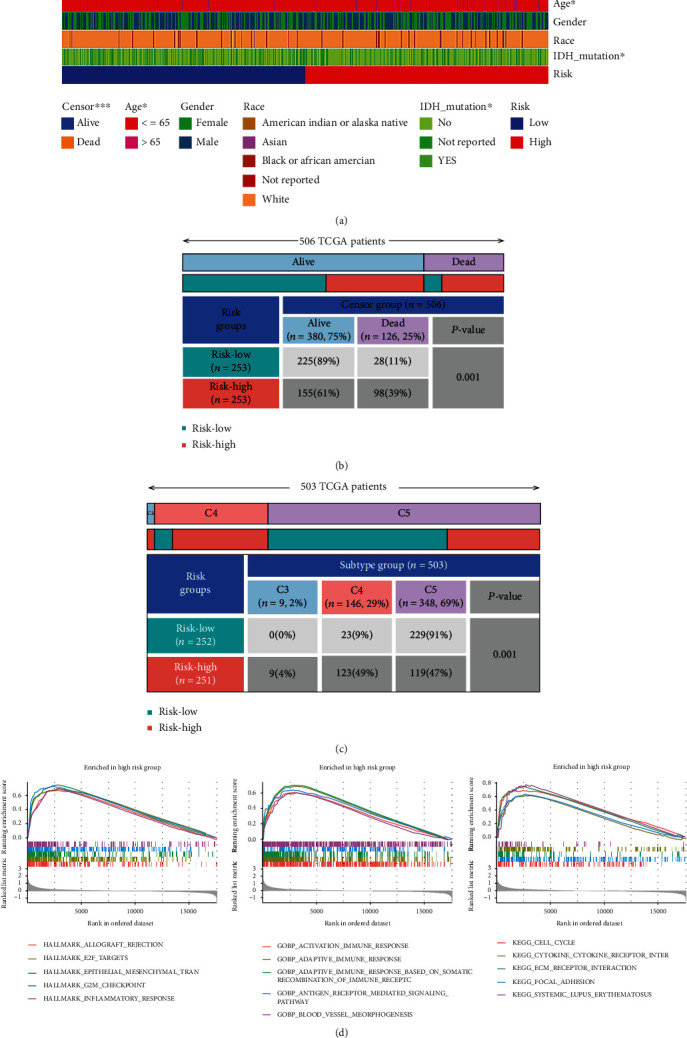
Identification of clinical feature between the low- and high-risk score groups: (a) Clinical risk analysis between the low- and high-risk groups; the patients in the high-risk group had a worse prognosis; (b, c) the *χ*^2^ test of the relationship between patients' outcome and the risk group and between the immune subtype group and risk group and the worse outcome was significantly related to the high-risk group; the C3 immune subtype group was positively correlated with the high-risk group. (d) The GSEA of hallmark genes, GO pathway, and KEGG pathway.

According to the clinical risk analysis, patients in high-risk group tend to have a worse prognosis (*P* < 0.001) (Figure 6(a)), patients older than 65 and patients with IDH mutation were more likely to belong to the high-risk group (Figure 6(a)). The *χ*^2^ test of the relationship between patients' outcome and the risk group and the worse outcome was significantly related to the high-risk group (Figure 6(b)). As the *χ*^2^ test result showed (Figure 6(c)), the C3 immune subtype group of LGG was positively correlated with high risk, and the correlation was statistically significant (*P* < 0.001).

The GSEA of hallmark genes of cancer showed that allograft rejection, E2F targets, epithelial mesenchymal transition, G2M checkpoint, and inflammatory response were enriched in the high-risk group. Also, the GSEA of GO pathway displayed that adaptive immune response, adaptive immune response based on somatic recombination of immune receptor, antigen receptor-mediated signaling pathway, and blood vessel morphogenesis were enriched in the high-risk group. The GSEA of KEGG pathway analysis showed that cell cycle, cytokine-cytokine receptor interaction, ECM receptor interaction, focal adhesion, and systemic lupus erythematosus were enriched in the high-risk group (Figure 6(d)).

### 3.4. Infiltrating Immune Cells in LGG

**Figure 7:**
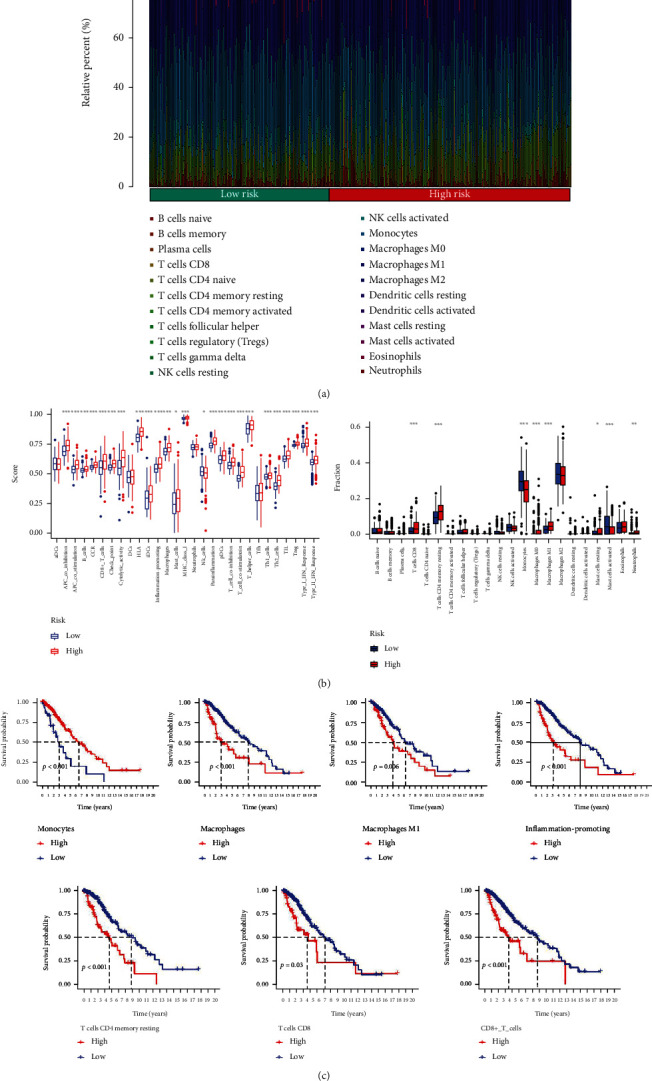
Identification of infiltrating immune cells in LGG and the relationship between immune cells and the risk group: (a) the distribution of 22 immune cells in LGG which was identified using CIBERSORT estimation; (b) the score of immune function and the fraction of immune cells between the high- and low-risk score groups; T cell CD8, CD4 memory resting T cells, and M0 macrophages. M1 macrophages had significantly higher fraction in the high-risk group (*P* < 0.001). Monocytes had lower faction in the high-risk group (*P* < 0.001); CD8+ T cells, inflammation promotion, macrophages, mast cells, T cell coinhibition, and T cell costimulation scored significantly higher in the high-risk group; macrophages, M1 macrophages, CD4 memory resting T cells, and T cell CD8 scored significantly high in the low-risk group; (c) the overall survival analysis of monocytes, macrophages, M1 macrophages, inflammation promotion, CD4 memory resting T cells, T cell CD8, and CD8+_T_cells between the high- and low-risk score groups; the higher fraction of macrophages, M1 macrophages, CD4 memory resting T cells, and T cell CD8 in LGG was associated with worse clinical prognosis, and the higher faction of monocytes in LGG was associated with better clinical prognosis. The higher score of CD8+ T cells and inflammation promotion in LGG was associated with worse clinical prognosis.

The results of distribution of 22 immune cells identified by CIBERSORT estimation are displayed in Figure 7(a); several immune cells, including CD8 T cells, CD4 memory resting T cells, M0 macrophages, and M1 macrophages had a significantly higher fraction in the high-risk group (*P* < 0.001) (Figure 7(b)). Specifically, monocytes had a lower fraction in the high-risk group (*P* < 0.001). For immune function, lots of that scored significantly higher in the high-risk group, which includes CD8+ T cells, inflammation promotion, macrophages, mast cells, T cell coinhibition, and T cell costimulation (Figure 7(b)). Furthermore, the survival analysis showed that the higher fraction of macrophages, M1 macrophages, CD4 memory resting T cells, and CD8 T cells in LGG was associated with worse clinical prognosis, and the higher fraction of monocytes in LGG was associated with better clinical prognosis. The higher score of CD8+ T cells and inflammation promotion in LGG was associated with worse clinical prognosis (Figure 7(c)).

### 3.5. Identification of Potential Signal Axis

**Figure 8:**
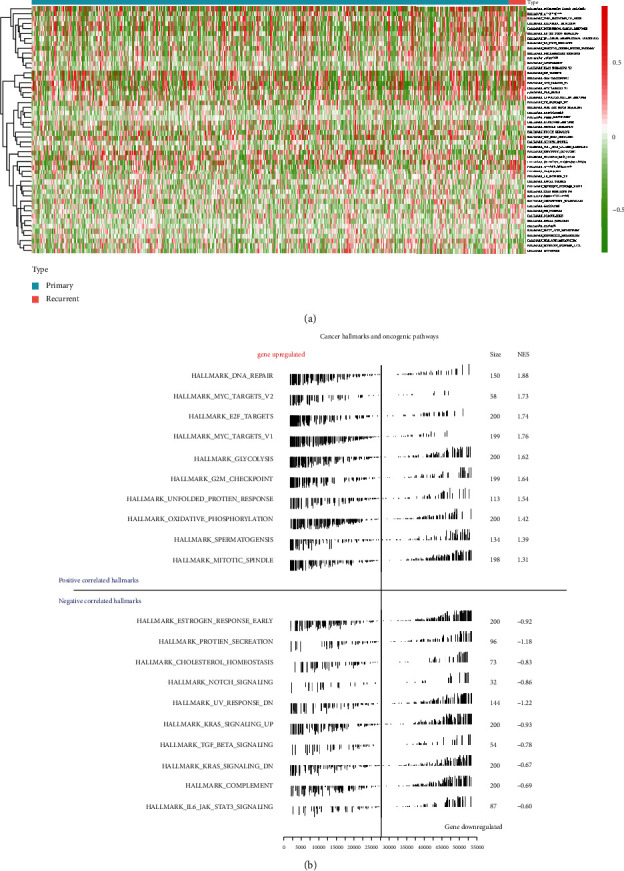
The expression levels of hallmark gene sets in primary and recurrent LGG. (a) The heat map plot showed the expression levels of 50 hallmark gene sets in primary and recurrent LGG. (b) The quantification of hallmark gene set expression using GSVA; 46 significantly expressed hallmark gene sets were screened out.

**Figure 9:**
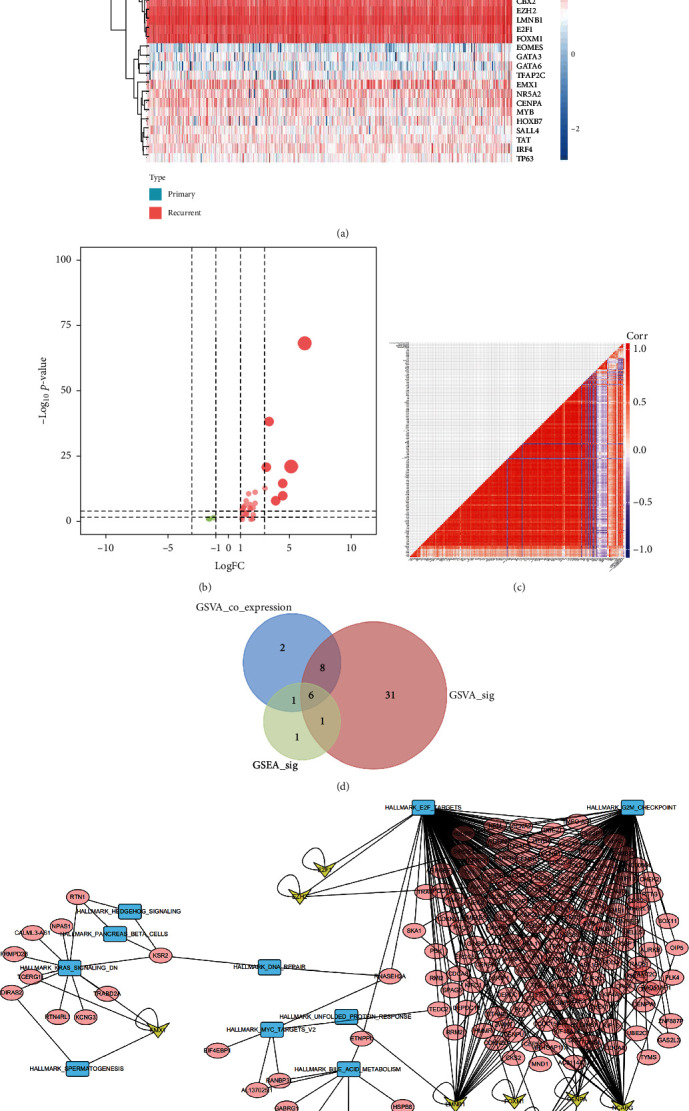
(a) The heat map showed the expression level of 38 differently expressed TFs in primary and recurrent LGG. (b) The volcano plot showed different expressed genes of 38 TFs. (c) The heat map showed the coanalysis for key TFs, prognostic genes, and hallmark gene sets.(d) The Venn plot showed the result of the intersection model among GSVA, GSEA, and WGCNA. (e) A regulated network for 8 TFs, prognostic key genes, and 10 hallmark gene sets.

Results of hallmark gene set expression levels were presented in a heat map plot (Figure 8(a)). The result of the quantification of gene expression of hallmark gene sets by GSVA is displayed in Figure 8(b), and 46 significantly expressed hallmark gene sets were screened out. 32 significantly differential expressed TFs were identified based on coexpression analysis, and the results were shown in the heat map (Figure 9(a)) and the volcano plot (Figure 9(b)). The heat map in Figure 9(c) displayed the coanalysis results for those key TFs, prognostic genes, and hallmark gene sets. An intersection model was constructed within the GSVA, GSEA, and WGCNA, and the result was shown in the Venn plot (Figure 9(d)). What is more, a network for TFs, prognostic key genes, and hallmark gene sets was constructed (Figure 9(e)). The most significant TF-DEG pair was NCAPG-AURKA (correlation coefficient = 0.914, *P* < 0.001), and the DEG-hallmark gene set pair was AURKA-E2F targets (correlation coefficient = 0.669, *P* < 0.001).

Therefore, a scientific hypothesis was put forward: AURKA was upregulated by NCAPG, then promoting the E2F target pathway, which might be a key biological mechanism in LGG recurrence.

### 3.6. ATAC-seq and ChIP-seq Validation

**Figure 10:**
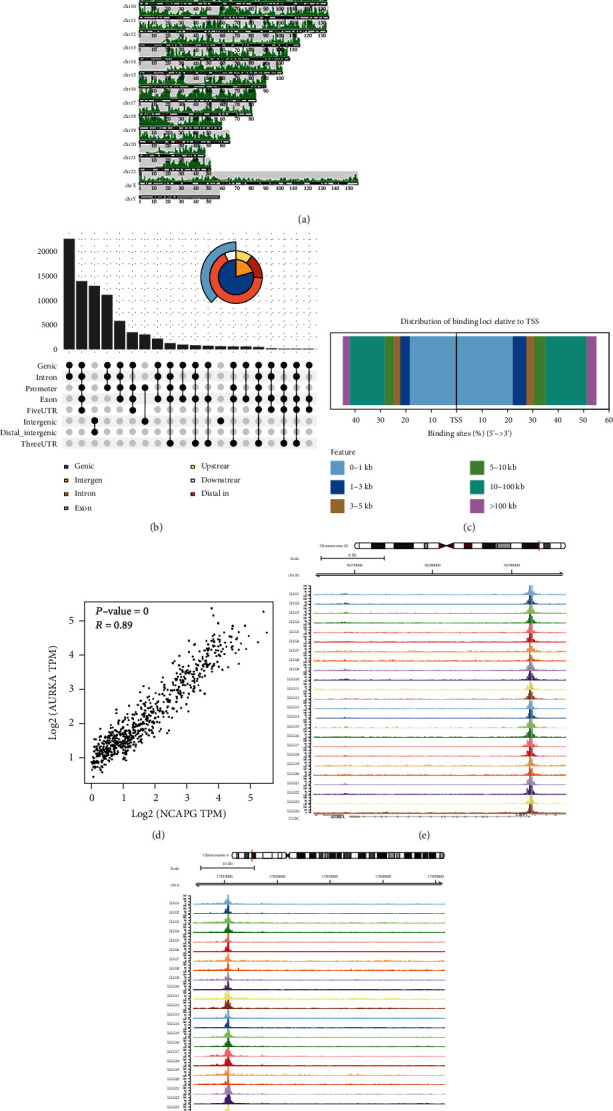
ATAC-seq validation: (a) identification of open chromatin loci in 24 chromosomes of LGG cells; each picks represented an open chromatin loci; (b) identification of pick types (including genic, intergenic, exon, upstream, intron, and distal intergenic); (c) the distribution of binding loci according to transcription start sites (TSS); (d) the transcripts per million (TPM) of AURKA and NCAPG had a notably positive correlation (*R* = 0.89, *P* < 0.001); (e, f) the strong binding peaks of AURKA and NCAPG were found in LGG.

**Figure 11:**
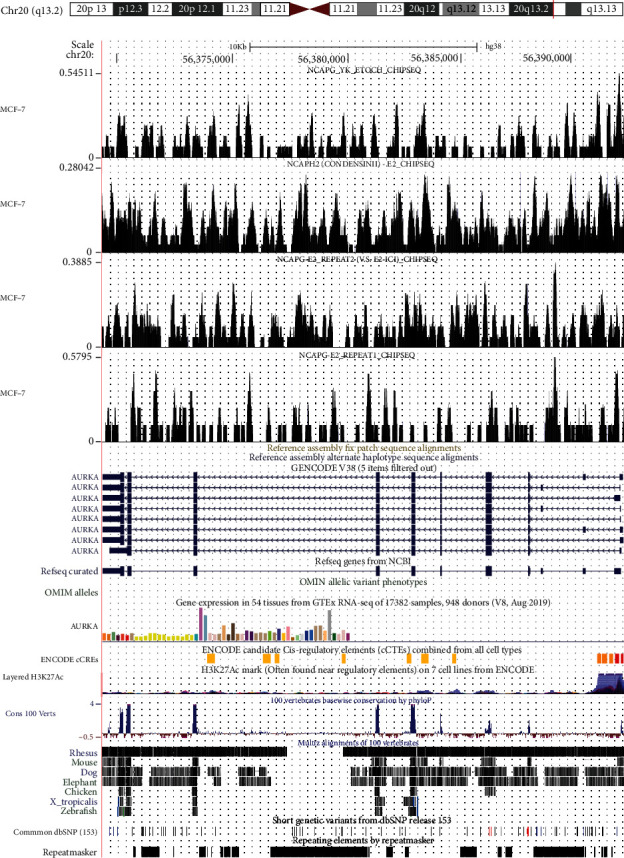
ChIP-seq result revealed the directly binding relationship of DNA fragment between AURKA and NCAPG.

Multiple open chromatin loci on different chromosomes of LGG cells were identified (Figure 10(a)), and the promoter area took the largest part (Figure 10(b)). Also, the distribution of binding loci according to transcription start sites (TSS) was displayed (Figure 10(c)). The transcripts per million (TPM) of AURKA and NCAPG had a notably positive correlation (*R* = 0.89, *P* < 0.001) (Figure 10(d)), and strong ATAC-seq binding peaks of AURKA and NCAPG in LGG cells were found (Figures 10(e) and 10(f)), indicating that those regions may act as potential interactive areas and that NCAPG upregulates AURKA and influences the biological behavior of LGG cells. Furthermore, the ChIP-seq results revealed the directly binding relationship of DNA fragment between AURKA and NCAPG (Figure 11).

### 3.7. Single-Cell RNA Sequencing Validation

**Figure 12:**
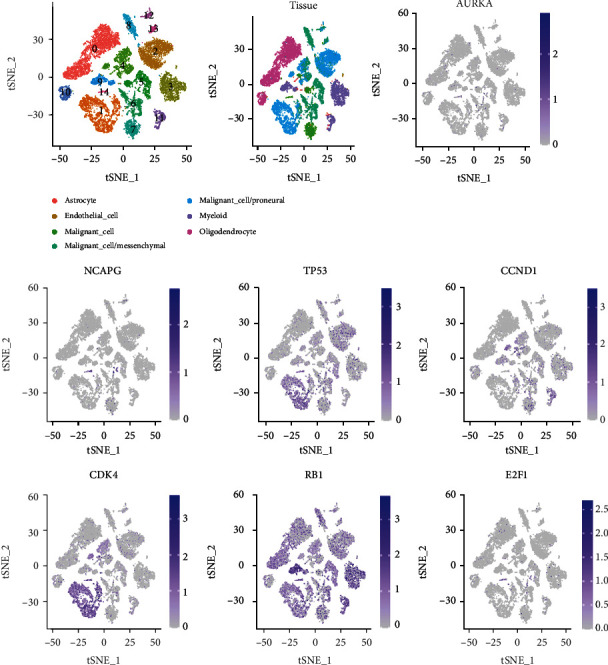
Single-cell RNA sequencing validation to detect the expression pattern of AURKA, NCAPG, TP53, CCND1, CDK4, RB1, and E2F1 in the cellular level; those genes expressed in 7 cell types including astrocyte, endothelial cell, malignant cell, malignant cell/mesenchymal, malignant cell/proneural, myeloid, and oligodendrocyte. CDK4 and TP53 are highly expressed in malignant cell/proneural; RB1 is expressed in all 7 cell types, especially highly expressed in myeloid.

We took the single-cell sequencing analysis to detect the expression pattern of AURKA, NCAPG, TP53, CCND1, CDK4, RB1, and E2F1 in the cellular level, and the results are displayed in Figure 12. The results showed that those genes were expressed in different levels in 7 cell types including astrocyte, endothelial cell, malignant cell, malignant cell/mesenchymal, malignant cell/proneural, myeloid, and oligodendrocyte. CDK4 and TP53 were highly expressed in malignant cell/proneural, and RB1 was expressed in all 7 cell types, especially highly expressed in myeloid.

### 3.8. Immunohistochemistry Validation

**Figure 13:**
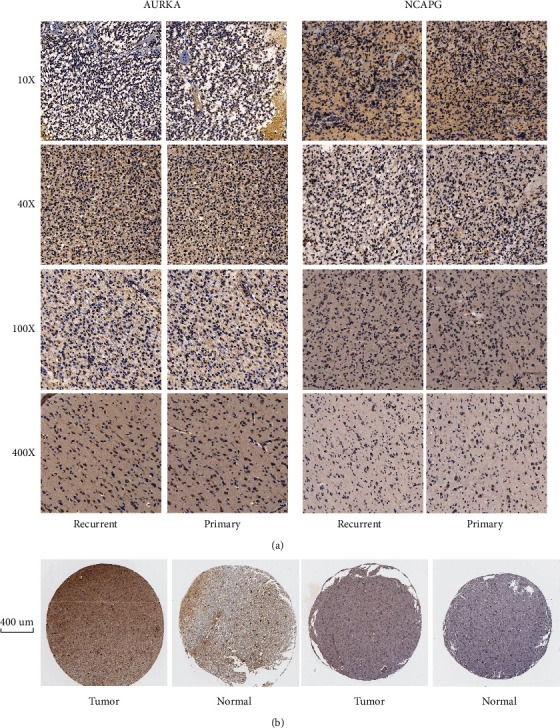
Immunohistochemical staining: (a) immunohistochemical analysis of AURKA and NCAPG expression in LGG specimens showing relatively higher expression in recurrent LGG samples. The Pearson correlation coefficient between AURKA and NCAPG was 0.642 (*P* < 0.001). (b) Immunohistochemical staining from the Human Protein Atlas: AURKA: normal tissues: glial cell staining: low, neuronal cell staining: low; tumor tissues: staining: low; NCAPG: normal tissues: endothelial cell staining: low, glial cell staining: not detected, neuronal cell staining: medium, neuropil staining: not detected; tumor tissues: staining: medium.

Immunohistochemical staining results (Figure 13) showed that AURKA and NCAPG expression levels in recurrent LGG patients were much higher than those in primary LGG patients, and the Pearson correlation coefficient between AURKA and NCAPG was 0.642 (*P* < 0.001).

### 3.9. Connectivity Map Analysis

**Figure 14:**
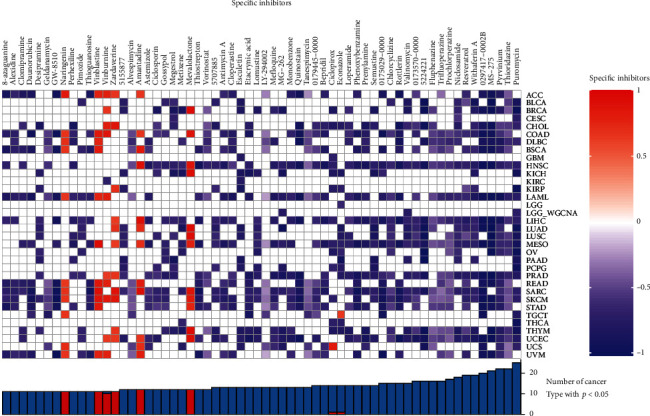
Connectivity Map analysis: the heat map showed that MG-262, valinomycin, and puromycin were significantly correlated with LGG.

To explore the connection between the potential axis and drug action, the CMap analysis was conducted. The heat map (Figure 14) showed that MG-262, valinomycin, and puromycin could be promising therapeutic agents in LGG.

### 3.10. Multidimensional Validation

Based on GeneCards, the top five genes in E2F targets were E2F1, TP53, RB1, CCND1, and CDK4. The summarized results of multidimensional validation are displayed in Table 2, and detailed information is displayed in Figures S1–S5.

AURKA as the stemness-related genes and its upstream transcription factor NCAPG were proven to have a higher expression level in tumor samples than normal sample in the Oncomine database (Figure S2). The higher expression levels of AURKA (Figure S3, *P* < 0.001; Figure S5, *P* < 0.001) and NCAPG (Figure S3, *P* < 0.001; Figure S5, *P* < 0.001) were also associated with higher grades in LGG, which was proven by UALCAN (Figure S3) and TISIDB (Figure S5). From the clinical level, AURKA (Figure S1H, HR (high) = 2.6, *P* < 0.001; Figure S3, *P* < 0.001; Figure S4, *P* < 0.001; Figure S5, *P* < 0.001) and NCAPG (Figure S1G, HR (high) = 2.9, *P* < 0.001; Figure S3, *P* < 0.001; Figure S4, *P* < 0.001; Figure S5, *P* < 0.001) showed a positive correlation with worse clinical outcome in the results form GEPIA (Figure S1), UALCAN (Figure S3), LinkedOmics (Figure S4), and TISIDB (Figure S5).

The top five genes in the downstream pathway were also identified to have a close association with LGG. In the GEPIA and Oncomine database, TP53 (Figure S1D, Figure S2), RB1 (Figure S1E, Figure S2), CCND1 (Figure S1F, Figure S2), and CDK4 (Figure S1G, Figure S2) showed a higher expression level in tumor samples than normal samples in LGG. The UALCAN and TISIDB databases have proven that the higher expression of E2F1 (Figure S3, *P* < 0.001; Figure S5, *P* < 0.001), TP53 (Figure S3, *P* < 0.001; Figure S5, *P* < 0.001), RB1 (Figure S3, *P* < 0.001; Figure S5, *P* < 0.001), CCND1 (Figure S3, *P* < 0.001; Figure S5, *P* < 0.001), and CDK4 (Figure S3, *P* < 0.001; Figure S5, *P* < 0.001) was also related to higher grade in LGG. What is more, in GEPIA, UALCAN, LinkedOmics, and TISIDB databases, E2F1 (Figure S1I, HR (high) = 2.9, *P* = 0.0014; Figure S3, *P* < 0.001; Figure S4, *P* < 0.001; Figure S5, *P* < 0.001) showed a positive correlation with worse clinical outcome. As for TP53 and RB1, data form GEPIA and LinkedOmics showed that TP53 (Figure S1J, HR (high) = 1.6, *P* = 0.0072; Figure S4, *P* < 0.001), RB1 (Figure S1K, HR (high) = 1.7, *P* = 0.0025; Figure S4, *P* < 0.001) had a positive correlation with worse clinical outcome, and other databases did not show obvious relation between these two genes and clinical outcome in LGG. For CCND1 and CDK4, data from UALCAN, LinkedOmics, and TISIDB showed that CCND1 (Figure S3, *P* = 0.002; Figure S4, *P* < 0.001; Figure S5, *P* < 0.001), and CDK4 (Figure S3, *P* = 0.002; Figure S4, *P* < 0.001; Figure S5, *P* < 0.001) had a positive correlation with worse clinical outcome.

## 4. Discussion

LGG is an important group of primary tumors in the CNS that mainly affects young people aged 20-40 years old [5]. They account for about 3%-15% among all brain tumors [5]. LGG was classified into several groups like astrocytoma, oligoastrocytoma, and oligodendroglioma, based on their histologic features [48]. LGG often grows slowly. Patients with LGG usually have long-term neurological symptoms, and more than 80% suffer seizures. However, it is worth mentioning that some patients with LGG do not have any symptoms [5]. Despite its slow growth and a long lifetime, most of patients will suffer recurrence and metastasis [6]. Previous studies have proven the importance of the alteration of IDH1/2 and chromosomes 1 and 19 in long-term survival and a high recurrence rate [8, 9]. But the specific biological mechanism is still unclear, and that makes clinical treatment tough. Therefore, further researches are very necessary.

In this study, differences between primary and recurrent LGG samples were analyzed to find out the potential regulation mechanism of LGG recurrence and provide new clues for future researches and clinical practice. Univariate and multivariate Cox proportional hazard regression analyses were utilized to screen out prognostic key genes. Pearson correlation analyses were conducted to select significantly correlated TF and downstream signaling pathway. Then, a recurrence-specific network was constructed and a potential regulatory pathway was identified: NCAPG worked as TF upregulating prognostic key gene AURKA and drove E2F target pathway. Single-cell sequencing analysis and immunohistochemistry validated the expression of AURKA and NCAPG on a cellular level and histological level, respectively. What is more, several online databases were used to testify the accuracy of the scientific hypothesis. Based on data in ChIP-Atlas and the results of ATAC-seq analysis, AURKA might be a potential target gene of NCAPG. Besides, the STRING database also showed a potential relationship between NCAPG and AURKA. According to Oncomine, NCAPG, AURKA, TP53, RB1, CCND1, and CDK4 were upregulated in tumor samples compared to paratumor samples. UALCAN and TISIDB showed that the higher expression of all these 7 genes was associated with a higher grade of LGG. Moreover, based on several online databases, upregulated expression of these 7 genes all displayed high correlation with worse clinical outcome of patients with LGG.

Non-SMC Condensin I Complex Subunit G (NCAPG) is a chromosomal condensing protein related to mitosis [49] and was encoded by NY-MEL-3 gene located on chromosome 4p [50]. A previous study has shown that NCAPG expression level was much higher in pediatric high-grade gliomas (pHGG) than in pediatric LGG (pLGG), and the knockdown of NCAPG slowed cell proliferation, which has indicated that NCAPG play an important role in gliomagenesis [51].

As a mitotic-related serine/threonine kinase, aurora A (AURKA) plays a key role in both mitosis and nonmitosis [52]. The abnormal expression of AURKA has been associated with a number of cancers [53]. The top five genes in the key hallmark gene sets were E2F1, TP53, RB1, CCND1, and CDK4; all of those genes play a prominent part in cell cycle and are closely related to amounts of tumors, such as prostate cancer, breast cancer, sarcomas, and brain tumors [54–57]. However, how NCAPG, AURKA, and the downstream pathway interact and how that causes LGG recurrence are still unclear.

Immunity plays a crucial role in therapy and prognosis in tumor management [58]. In the tumor, six immune subtypes, including C1-C6, were identified [59]. Our results showed that the C3 immune subtype (inflammatory, defined by higher expression of Th1 and Th17 genes) of LGG was positively related to the high-risk group. According to the GSEA of GO pathway, inflammatory response was enriched in patients in the high-risk group, and the GSEA of KEGG pathway showed that systematic lupus erythematosus was related to high-risk scores. What is more, the results of CIBERSORT estimation showed that the scores of CD8+ T cells and inflammation promoting were significantly higher in the high-risk group than those in the low-risk group. Overall, it is reasonable to assume that the recurrence of LGG was related to an inflammatory response conducted by CD8+ T cells. Besides, autoimmune diseases could trigger chronic inflammation, which was associated with developing cancer [60], and Th17 produced by activated CD8+ T cells played a crucial role in the development of autoimmunity disease [61]; therefore, autoimmune may be a potential mechanism in the recurrence of LGG.

By using CMap analysis, MG-262, valinomycin, and puromycin were screened out as specific inhibitors of the key pathway. Particularly, puromycin is a protein synthesis inhibitor, and its structure is similar to tyrosyl-tRNA and could interfere translation process [24]. Puromycin has been proven its antibiotic properties, and it also could be used in cancer diagnosis and treatment [25]. Previous research has shown the antitumor effects against leukemic cell lines in vitro of puromycin and its analogs [26]. Besides, puromycin proved to promote the function of farnesiferol c in downregulating CCND1 and CDK4 in non-small-cell lung cancer cells [62], which not only supports the scientific hypothesis but also provides a new clue for LGG treatments.

However, there are still some limitations in our researches that should be mentioned. Firstly, although the total sample size was sufficient, the number of recurrent samples was not enough, which meant some DEGs might not have been found. Secondly, genetic data related to LGG recurrence were insufficient in online databases, which limited the further multidimensional validation. What is more, despite the strict selective criteria in WGCNA and multidimensional analysis, error cannot be avoided. Last but not least, the scientific hypothesis has been validated only from bioinformatics level and there is still a long way to clinical practices. Therefore, experiments in molecular, cellular, and individual levels should be done in future studies.

## 5. Conclusion

Since the present information is not enough to understand the genetic regulation mechanism of biological characteristics like recurrence and how that affects clinical prognosis, therefore, we conducted our study. In summary, we constructed a specific regulatory network based on stemness-related genes to predict the potential biological mechanism in LGG recurrence. We proposed that AURKA, positively regulated by NCAPG, promoted E2F target pathway in LGG, which played an important role in LGG recurrence. And puromycin might be a specific inhibitor of the NCAPG-AURKA-E2F signal pathway. Besides, we also figured out that an inflammatory response conducted by CD8+ T cells might play a role in the recurrence of LGG. However, there are still some limitations in our researches such as insufficient sample size, insufficient genetic data, and lack of wet experiments. Therefore, experiments in molecular, cellular, and individual levels should be done in future studies.

## Figures and Tables

**Table 1:** Clinical data in LGG.

Variables	Total patients (*N* = 536)
Age (years)	
Mean ± SD	42.67 ± 13.32
Median (range)	40.00 (14-87)
Gender	
Female	242 (45.23%)
Male	293 (54.77%)
Race	
American Indian or Alaska Native	1 (0.18%)
Asian	8 (1.50%)
Black or African American	22 (4.11%)
Not reported	10 (1.87%)
White	494 (92.34%)
Future time (days)	
Mean ± SD	1004.83 ± 990.15
Median (range)	714 (1–6423)
NA	5
Primary or recurrent	
Primary	516 (96.27%)
Recurrent	20 (3.73%)
IDH1_mutation	
Yes	127 (23.69%)
No	315 (58.77%)
NA	94 (17.54%)

Abbreviations: SD: standard deviation; NA: not available.

**(a):** 

	NCAPG	AURKA	E2F1	TP53	RB1
GEPIA	Expression: - Survival: HR (high) = 2.9, *P* < 0.001	Expression: - Survival: HR (high) = 2.6, *P* < 0.001	Expression: - Survival: HR (high) = 1.8, *P* = 0.0014	Expression: high Survival: HR (high) = 1.6, *P* = 0.0072	Expression: high Survival: HR (high) = 1.7, *P* = 0.0052
Oncomine	Expression: high	Expression: high	Expression: NA	Expression: high	Expression: high
cBioPortal	Survival: *P* < 0.001	Survival: *P* = 0.289	Survival: *P* = 0.901	Survival: *P* = 0.034	Survival: *P* = 0.733
UALCAN	Grade: *P* < 0.001 Survival: *P* < 0.001	Grade: *P* < 0.001 Survival: *P* < 0.001	Grade: *P* < 0.001 Survival: *P* < 0.001	Grade: *P* < 0.001 Survival: *P* = 0.024	Grade: *P* < 0.001 Survival: *P* = 0.79
LinkedOmics	Survival: *P* < 0.001	Survival: *P* < 0.001	Survival: *P* < 0.001	Survival: *P* < 0.001	Survival: *P* < 0.001
TISIDB	Survival: *P* < 0.001 Grade: rho = 0.415, *P* < 0.001	Survival: *P* < 0.001 Grade: rho = 0.44, *P* < 0.001	Survival: *P* < 0.001 Grade: rho = 0.298, *P* < 0.001	Survival: *P* = 0.169 Grade: rho = 0.192, *P* < 0.001	Survival: *P* = 0.002 Grade: rho = 0.147, *P* < 0.001

**(b):** 

CCND1	CDK4	Results
Expression: high Survival: HR (high) = 1.3, *P* = 0.22	Expression: high Survival: HR (high) = 1.1, *P* = 0.59	TP53, RB1, CCND1, and CDK4 are significantly upregulated in LGG. The high expression of NCAPG, AURKA, E2F1, T53, and RB1 is correlated with worse prognosis in LGG (Figure S1).
Expression: high	Expression: high	NCAPG, AUTKA, TP53, RB1, CCND1, and CDK4 are significantly upregulated in LGG (Figure S2).
Survival: *P* = 0.687	Survival: *P* < 0.001	The high expression of NCAPG, TP53, and CDK4 is correlated with worse prognosis in LGG (Figure S3).
Grade: *P* < 0.001 Survival: *P* = 0.0021	Grade: *P* < 0.001 Survival: *P* = 0.0021	The high expression of NCAPG, AURKA, E2F1, TP53, and CCND1 is correlated with higher grade in LGG. NCAPG, AUTKA, E2F1, TP53, CCND1, and CDK4 are significantly upregulated in LGG (Figure S4).
Survival: *P* < 0.001	Survival: *P* < 0.001	The high expression of NCAPG, AURKA, E2F1, TP53, CCND1, and CDK4 is correlated with worse prognosis in LGG (Figure S5).
Survival: *P* = 0.805 Grade: rho = 0.204, *P* < 0.001	Survival: *P* = 0.497 Grade: rho = 0.204, *P* < 0.001	The high expression of NCAPG, AURKA, and E2F1 is correlated with worse prognosis in LGG. The high expression of NCAPG, AURKA, E2F1, TP53, RB1, CCND1, and CDK4 is correlated with higher grade in LGG (Figure S6).

## Data Availability

Data for analysis were from TCGA database and GEO database (https://www.ncbi.nlm.nih.gov/geo/query/acc.cgi?acc=GSE164041).

## Supplementary Materials

Supplementary Materials Figure S1: the external validation from Gene Expression Profiling Interactive Analysis (GEPIA): TP53, RB1, CCND1, and CKD4 had a higher expression level in tumor samples than in normal samples in LGG patients. The higher expression level of NCAPG, AURKA, E2F1, TP53, and RB1 was significantly related to worse clinical outcome in patients with LGG. Figure S2: the external validation from Oncomine: NCAPG, AURKA, TP53, RB1, CCND1, and CDK2 had a higher expression level in tumor samples than normal samples in LGG patients. Figure S3: the external validation from UALCAN: (A) the higher expression of NCAPG, AURKA, E2F1, TP53, RB1, CCND1, and CDK4 was related to higher grade in LGG; (B) NCAPG, AURKA, E2F1, CCND1, and CDK4 were related with worse clinical outcome. Figure S4: the external validation from LinkedOmics: the higher expression level of NCAPG, AURKA, E2F1, TP53, RB1, CCND1, and CDK4 was related to worse clinical outcome. Figure S5: the external validation from TISBID: (A) the higher expression level of NCAPG, AURKA, E2F1, CCND1, and CDK4 was related to worse clinical outcome; (B) the higher expression of NCAPG, AURKA, E2F1, TP53, RB1, CCND1, and CDK4 was related to higher grade in LGG. List of differentially expressed genes (DEGs).
