# Supplementary material for: The Recurrent-Specific Regulation Network of Prognostic Stemness-Related Signatures in Low-Grade Glioma
Source: Dis Markers. 2023 Jan 17;2023:2243928. doi: 10.1155/2023/2243928 (PMC9873439; doi:10.1155/2023/2243928)
Supplement: Supplementary Materials — Figure S1: the external validation from Gene Expression Profiling Interactive Analysis (GEPIA): TP53, RB1, CCND1, and CKD4 had a higher expression level in tumor samples than in normal samples in LGG patients. The higher expression level of NCAPG, AURKA, E2F1, TP53, and RB1 was significantly related to worse clinical outcome in patients with LGG. Figure S2: the external validation from Oncomine: NCAPG, AURKA, TP53, RB1, CCND1, and CDK2 had a higher expression level in tumor samples than normal samples in LGG patients. Figure S3: the external validation from UALCAN: (A) the higher expression of NCAPG, AURKA, E2F1, TP53, RB1, CCND1, and CDK4 was related to higher grade in LGG; (B) NCAPG, AURKA, E2F1, CCND1, and CDK4 were related with worse clinical outcome. Figure S4: the external validation from LinkedOmics: the higher expression level of NCAPG, AURKA, E2F1, TP53, RB1, CCND1, and CDK4 was related to worse clinical outcome. Figure S5: the external validation from TISBID: (A) the higher expression level of NCAPG, AURKA, E2F1, CCND1, and CDK4 was related to worse clinical outcome; (B) the higher expression of NCAPG, AURKA, E2F1, TP53, RB1, CCND1, and CDK4 was related to higher grade in LGG. List of differentially expressed genes (DEGs). [file 2243928.f1.zip › List of differentially expressed genes.docx]

**List of differentially expressed genes (DEGs)**

| gene | logFC | entrezID |
| --- | --- | --- |
| AP006284.1 | 7.043147751 | NA |
| LMNTD2 | 5.75155622 | 256329 |
| DNM3OS | 4.709728045 | 100628315 |
| LINC02158 | 5.025060528 | 729083 |
| AC092198.1 | 6.42425723 | NA |
| RASSF7 | 3.432724733 | 8045 |
| COL2A1 | 6.658116911 | 1280 |
| MUC6 | 3.84113984 | 4588 |
| PAX2 | 5.955462068 | 5076 |
| HOXB9 | 7.565232431 | 3219 |
| ONECUT3 | 5.200906428 | 390874 |
| GCGR | 5.790102785 | 2642 |
| SLC6A3 | 6.444958499 | 6531 |
| CDX2 | 6.189075477 | 1045 |
| IRX4 | 5.794217941 | 50805 |
| CCL17 | 3.682599294 | 6361 |
| RSPO1 | 4.107659072 | 284654 |
| AC008443.4 | 3.763310505 | NA |
| SLC6A10P | 5.372003643 | 386757 |
| ALOX15 | 5.055861784 | 246 |
| AC133561.1 | 4.22111513 | NA |
| FZD10 | 3.389376206 | 11211 |
| CCDC144NL-AS1 | 4.017145625 | 440416 |
| TBX1 | 4.126174096 | 6899 |
| ANKRD19P | 2.646867335 | 138649 |
| WNT3A | 5.259968269 | 89780 |
| TPSG1 | 4.269127298 | 25823 |
| BNC1 | 4.290802247 | 646 |
| MEOX1 | 4.499792992 | 4222 |
| SIX2 | 4.09192361 | 10736 |
| GALR2 | 3.718378694 | 8811 |
| SLC18A2 | 2.987966967 | 6571 |
| PLA2G2D | 4.214829917 | 26279 |
| HOXC12 | 8.34209919 | 3228 |
| ISL2 | 4.944551277 | 64843 |
| AC022905.1 | 4.620873978 | NA |
| FENDRR | 2.9169264 | 400550 |
| HES2 | 4.369765221 | 54626 |
| BARX1 | 5.434224603 | 56033 |
| HS3ST6 | 4.978149388 | 64711 |
| DMBX1 | 5.654126817 | 127343 |
| LBX1 | 6.533985532 | 10660 |
| CD1B | 5.263005394 | 910 |
| HMX3 | 6.323964104 | 340784 |
| AL132712.1 | 3.93652608 | NA |
| FAR2P1 | 4.350105505 | 440905 |
| AC027243.1 | 5.299285171 | NA |
| KCNA7 | 3.281768579 | 3743 |
| AC120498.10 | 4.20514785 | NA |
| AC012354.1 | 4.215342424 | NA |
| MAFA | 4.701252882 | 389692 |
| TWIST2 | 3.360644763 | 117581 |
| CRABP2 | 2.895234954 | 1382 |
| CCL22 | 3.350594346 | 6367 |
| AL136981.1 | 5.088358353 | NA |
| SIM1 | 6.015091999 | 6492 |
| C15orf48 | 3.331330307 | 84419 |
| DLX3 | 3.928694212 | 1747 |
| ISM1 | 2.788003757 | 140862 |
| IRF4 | 3.307776076 | 3662 |
| SDR42E1 | 3.813657305 | 93517 |
| IGF2 | 4.016750746 | 3481 |
| HIST1H3H | 3.184087501 | NA |
| PRAC1 | 6.689838432 | 84366 |
| HES7 | 2.921855554 | 84667 |
| WNT11 | 2.671121747 | 7481 |
| AC126175.2 | 4.21877165 | NA |
| ESPN | 3.815874668 | 83715 |
| MMP12 | 5.700331537 | 4321 |
| PRAC2 | 6.062018436 | 360205 |
| TRIM7 | 2.517732328 | 81786 |
| LBX1-AS1 | 5.59138187 | 399806 |
| ECEL1 | 4.613834688 | 9427 |
| GUSBP6 | 4.618792755 | 653435 |
| RIPPLY3 | 3.854035527 | 53820 |
| IGF2-AS | 4.891844309 | 51214 |
| NTF3 | 4.149664387 | 4908 |
| LINC02754 | 4.576425451 | 105369360 |
| GBX1 | 2.970479422 | 2636 |
| CCL13 | 4.466057468 | 6357 |
| DPT | 3.4153464 | 1805 |
| AC012123.1 | 4.433114864 | NA |
| ALX4 | 3.68449281 | 60529 |
| AC008060.3 | 5.109044096 | NA |
| HIST1H2AI | 3.690107677 | NA |
| ST8SIA6-AS1 | 3.336902551 | 100128098 |
| GEN1 | 1.940284566 | 348654 |
| TCL6 | 3.511527067 | 27004 |
| BHLHE23 | 5.972864432 | 128408 |
| PRRX2 | 3.918498033 | 51450 |
| EN2 | 3.373109487 | 2020 |
| PRAME | 5.763322357 | 23532 |
| AC027243.2 | 4.792045844 | NA |
| KC877982.1 | 4.757641697 | NA |
| WNT6 | 2.786989014 | 7475 |
| COL1A2 | 3.261167311 | 1278 |
| GPR50 | 4.683318942 | 9248 |
| PITX1 | 3.98489097 | 5307 |
| EBF2 | 3.2079039 | 64641 |
| LBX2 | 2.475235956 | 85474 |
| SLC22A31 | 3.799605445 | 146429 |
| HOXB6 | 4.027185127 | 3216 |
| AL441963.1 | 2.455527327 | NA |
| FOXD2 | 3.063667685 | 2306 |
| AC078906.1 | 3.651468758 | NA |
| MAFA-AS1 | 4.725920438 | 104326051 |
| AP001476.1 | 3.615524337 | NA |
| ANKRD18DP | 2.912671458 | 348840 |
| AC132825.4 | 4.395856145 | NA |
| NCCRP1 | 3.382710561 | 342897 |
| FBN2 | 2.665538713 | 2201 |
| CD70 | 4.145277247 | 970 |
| KAZALD1 | 2.432081597 | 81621 |
| TBX21 | 2.68894323 | 30009 |
| ADAMTS7 | 2.661656737 | 11173 |
| TBX3 | 1.966311029 | 6926 |
| HSD11B2 | 2.080881621 | 3291 |
| ISYNA1 | 1.75940222 | 51477 |
| HMX2 | 5.181144593 | 3167 |
| CILP2 | 2.282870929 | 148113 |
| GLT8D2 | 1.720220223 | 83468 |
| LINC01694 | 2.865735573 | 105372840 |
| PAX7 | 4.038543461 | 5081 |
| NHLH1 | 2.655927636 | 4807 |
| KLHL14 | 3.199005121 | 57565 |
| FBP1 | 2.342944179 | 2203 |
| AC073488.1 | 4.817206744 | NA |
| ANHX | 4.622806828 | 647589 |
| EBF3 | 3.210026555 | 253738 |
| DNAH8 | 2.52349404 | 1769 |
| PRDM6 | 1.999328319 | 93166 |
| LRRC15 | 3.770733808 | 131578 |
| FOXA2 | 5.10316809 | 3170 |
| TFAP2C | 3.081755396 | 7022 |
| COL5A1 | 3.062287605 | 1289 |
| HOXC-AS3 | 4.975009812 | 100874365 |
| ADAMTS2 | 2.351528409 | 9509 |
| LINC01305 | 3.15891557 | 285084 |
| TFF3 | 3.753496075 | 7033 |
| LHX4 | 1.822461635 | 89884 |
| SHISA2 | 2.521343238 | 387914 |
| FZD2 | 2.094403605 | 2535 |
| MMP11 | 2.353249982 | 4320 |
| MFAP2 | 3.068546891 | 4237 |
| DSP | 2.080371828 | 1832 |
| NID2 | 2.399602923 | 22795 |
| AP003068.4 | 1.86713829 | NA |
| CA6 | 3.325341992 | 765 |
| CGN | 1.32845256 | 57530 |
| CD248 | 2.580311099 | 57124 |
| TBXT | 3.737695289 | 6862 |
| TLX3 | 4.602518065 | 30012 |
| NKD2 | 1.887049518 | 85409 |
| NKX3-2 | 3.272904624 | 579 |
| AC005481.1 | 2.121443877 | NA |
| CGB7 | 3.050509743 | 94027 |
| COMP | 3.146385402 | 1311 |
| CD19 | 2.000297827 | 930 |
| AC138474.1 | 1.995821747 | NA |
| LMX1B | 3.327895468 | 4010 |
| LINC00871 | 3.936480599 | 100506412 |
| HIST1H2AG | 2.483625172 | NA |
| LRRC56 | 1.505049961 | 115399 |
| CAMP | 3.618509853 | 820 |
| DNAJC22 | 2.130217817 | 79962 |
| BCOR | 1.21989617 | 54880 |
| LRCOL1 | 2.122937768 | 100507055 |
| RPL38 | 1.176948568 | 6169 |
| SCX | 2.245239638 | 642658 |
| LGALS2 | 1.757368205 | 3957 |
| SCUBE1 | 2.673810852 | 80274 |
| AC012531.1 | 4.029827416 | NA |
| LINC01389 | 1.691220301 | 102724077 |
| COX6B2 | 2.079317923 | 125965 |
| MCTP2 | 2.544738339 | 55784 |
| HOXB13 | 4.416464295 | 10481 |
| B4GALNT3 | 2.006136464 | 283358 |
| SLC9A3 | 1.743111994 | 6550 |
| MTDHP1 | 1.646921404 | 100418814 |
| RPP25 | 1.967796686 | 54913 |
| CNPY1 | 2.561128695 | 285888 |
| PIK3R2 | 1.276600255 | 5296 |
| LIMD2 | 1.271217377 | 80774 |
| PAX9 | 2.279777842 | 5083 |
| PASK | 1.216878736 | 23178 |
| TRAIP | 1.396908983 | 10293 |
| AC010478.1 | 3.983369951 | NA |
| BANCR | 2.94354153 | 100885775 |
| NXPH4 | 2.190831168 | 11247 |
| AL035658.1 | 2.588338604 | NA |
| COL6A1 | 1.460139602 | 1291 |
| LINC02804 | 1.896995026 | 105371228 |
| AC015912.3 | 2.007925786 | NA |
| COL18A1 | 1.620174653 | 80781 |
| EMC9 | 1.030983226 | 51016 |
| AC091806.1 | 2.568631103 | NA |
| FOXC2 | 1.919510957 | 2303 |
| SLC4A11 | 1.863491556 | 83959 |
| SCUBE3 | 1.841402738 | 222663 |
| AC109635.3 | 3.532076108 | NA |
| RPL36A | 1.226663005 | 6173 |
| C1QL4 | 2.834773027 | 338761 |
| AL031058.1 | 2.138231385 | NA |
| COL6A2 | 2.545319841 | 1292 |
| NTSR1 | 2.912077477 | 4923 |
| ADAMTS17 | 1.687150075 | 170691 |
| JPT1 | 1.197366941 | 51155 |
| TEDC1 | 1.304977405 | 283643 |
| CD1C | 2.673821607 | 911 |
| PIWIL1 | 3.314740632 | 9271 |
| MNX1 | 2.93860616 | 3110 |
| ST8SIA6 | 1.617197292 | 338596 |
| GATA6 | 2.964800495 | 2627 |
| NPBWR1 | 3.812934235 | 2831 |
| AC209154.1 | 4.674706013 | NA |
| ADAMTS7P3 | 2.100904679 | 400406 |
| ZNF750 | 1.835038656 | 79755 |
| AC092171.5 | 1.329063528 | NA |
| SPC24 | 2.245630789 | 147841 |
| LTK | 2.256167977 | 4058 |
| ROR2 | 2.752690482 | 4920 |
| CCDC167 | 1.136977308 | 154467 |
| GAS2 | 1.602017352 | 2620 |
| MET | 3.016277079 | 4233 |
| EPS8L1 | 1.524340437 | 54869 |
| LYZ | 2.594218076 | 4069 |
| TEDC2 | 1.463617954 | 80178 |
| GATA6-AS1 | 3.431713662 | 100128893 |
| FOXF1 | 1.62895999 | 2294 |
| LINC02104 | 2.398609656 | 101926940 |
| ENPP1 | 1.926587578 | 5167 |
| SHISA8 | 2.255882283 | 440829 |
| TMEM114 | 3.074116476 | 283953 |
| SMOC2 | 1.560478817 | 64094 |
| COL15A1 | 2.297949398 | 1306 |
| HMGA2 | 3.870650283 | 8091 |
| AC068473.3 | 1.638522795 | NA |
| LINC01230 | 3.346236401 | 102800446 |
| HOXB5 | 3.848498733 | 3215 |
| MIR4635 | 2.397774261 | 100616479 |
| PCP4 | 2.420973956 | 5121 |
| SAC3D1 | 1.089495525 | 29901 |
| SNORD104 | 1.824338257 | 692227 |
| PURPL | 3.259168309 | 643401 |
| IL17RE | 1.203109469 | 132014 |
| EGFL6 | 2.473731141 | 25975 |
| TMEM200B | 1.560526913 | 399474 |
| AC046185.3 | 1.257458399 | NA |
| TNFSF9 | 1.797352134 | 8744 |
| SCML1 | 1.132679784 | 6322 |
| SALL4 | 2.165199335 | 57167 |
| SCN5A | 2.098930598 | 6331 |
| ELFN1 | 1.748622911 | 392617 |
| NPR3 | 2.483430329 | 4883 |
| NGFR | 2.379829799 | 4804 |
| RPL36 | 1.111667314 | 25873 |
| LINC01098 | 3.111735182 | 285501 |
| HIST2H2AC | 1.750140785 | NA |
| SFRP1 | 2.044820592 | 6422 |
| COL13A1 | 2.248468601 | 1305 |
| CKS1B | 1.065177877 | 1163 |
| FZD10-AS1 | 2.170511866 | 440119 |
| CBX2 | 1.636806623 | 84733 |
| RFLNB | 1.373987877 | 359845 |
| SNHG25 | 1.636918518 | 105376843 |
| TRDC | 2.850989325 | 28526 |
| PRR35 | 3.543776593 | 146325 |
| NXN | 1.08080973 | 64359 |
| NHS | 1.388344611 | 4810 |
| CCL18 | 3.70921971 | 6362 |
| SLC25A10 | 1.183395473 | 1468 |
| RPL27A | 1.04598459 | 6157 |
| VAX1 | 2.399369166 | 11023 |
| MRPL12 | 1.031729678 | 6182 |
| CDH23 | 1.574053386 | 64072 |
| CENPW | 1.491803771 | 387103 |
| RPL37P6 | 1.670617641 | 346950 |
| TEX11 | 2.38726248 | 56159 |
| MXD3 | 1.654164892 | 83463 |
| RAD51 | 1.781887419 | 5888 |
| CD1E | 2.896332609 | 913 |
| AC092614.1 | 2.040098103 | NA |
| FOXD2-AS1 | 1.979727923 | 84793 |
| SEMA3F | 1.721528606 | 6405 |
| HOXC11 | 4.42109069 | 3227 |
| COL1A1 | 2.779685253 | 1277 |
| BOLA2B | 1.180915925 | 654483 |
| MMP9 | 2.982124058 | 4318 |
| CDT1 | 1.667066245 | 81620 |
| EDARADD | 1.506534233 | 128178 |
| COL3A1 | 2.790343532 | 1281 |
| IL4I1 | 1.931255083 | 259307 |
| AC093866.1 | 2.68135342 | NA |
| CDCA4 | 1.221409373 | 55038 |
| AC025183.1 | 3.690383479 | NA |
| AC131212.4 | 1.934264208 | NA |
| PTTG1 | 1.861303389 | 9232 |
| AL133215.2 | 1.504774603 | NA |
| SP5 | 2.241601423 | 389058 |
| GATA2-AS1 | 1.594185007 | 101927167 |
| TLL2 | 1.655290167 | 7093 |
| HOXA11-AS | 3.621584914 | 221883 |
| CCR4 | 2.44419568 | 1233 |
| AL139339.2 | 1.642622504 | NA |
| MDK | 1.756671039 | 4192 |
| AP000251.1 | 1.403184111 | NA |
| SELP | 2.718148541 | 6403 |
| CACNA1H | 1.644504151 | 8912 |
| C1QTNF6 | 1.097875817 | 114904 |
| CTSK | 1.671843006 | 1513 |
| ATP2A1-AS1 | 1.311827538 | 100289092 |
| HOXC4 | 2.282692285 | 3221 |
| SDC1 | 1.885233319 | 6382 |
| AC005725.1 | 1.96227472 | NA |
| FAM83F | 1.717164081 | 113828 |
| UBE2T | 1.524033369 | 29089 |
| RFLNA | 1.340038381 | 144347 |
| FANCD2 | 1.437261488 | 2177 |
| SBSN | 2.380808722 | 374897 |
| MAD2L1 | 1.276305252 | 4085 |
| CD52 | 1.913702508 | 1043 |
| ZNF878 | 1.492681597 | 729747 |
| ISL1 | 3.984675977 | 3670 |
| AC090136.3 | 2.461937817 | NA |
| RECQL4 | 1.257941633 | 9401 |
| LINC01121 | 2.747627666 | 400952 |
| B3GALNT2 | 1.029756074 | 148789 |
| TONSL | 1.215062549 | 4796 |
| AC006487.1 | 1.965381828 | NA |
| LINC01740 | 2.108109061 | 101929565 |
| PAPOLB | 1.956957499 | 56903 |
| ADAMTS19 | 1.992629917 | 171019 |
| VASH2 | 1.551664975 | 79805 |
| NHLRC4 | 1.213185579 | 283948 |
| ODF3L2 | 1.683412942 | 284451 |
| CDC42EP5 | 1.563995812 | 148170 |
| AL133492.1 | 1.986088711 | NA |
| POLE | 1.037165783 | 5426 |
| RNASEH2A | 1.076329571 | 10535 |
| NT5DC2 | 1.013271581 | 64943 |
| SDK2 | 1.218384987 | 54549 |
| PRSS2 | 3.902389041 | 5645 |
| GDF6 | 2.078622738 | 392255 |
| PPP2R3B | 1.154563328 | 28227 |
| HAUS7 | 1.319305177 | 55559 |
| C2orf69P1 | 4.853073523 | 727964 |
| POC1A | 1.380786813 | 25886 |
| SPHK1 | 1.625025028 | 8877 |
| ACP5 | 2.044193887 | 54 |
| HAS3 | 1.205600524 | 3038 |
| OR3A2 | 1.970624685 | 4995 |
| AC004080.2 | 3.245607603 | NA |
| AC021723.1 | 1.828301942 | NA |
| NTRK1 | 1.68203772 | 4914 |
| CRABP1 | 2.706941712 | 1381 |
| FCRLA | 2.192142717 | 84824 |
| EIF4EBP1 | 1.211809542 | 1978 |
| BMX | 1.971901236 | 660 |
| SIX3 | 2.850191582 | 6496 |
| LINC01606 | 2.578705462 | 100507651 |
| AC006077.2 | 1.362481386 | NA |
| FAM43A | 1.202972084 | 131583 |
| PCOLCE | 1.732559834 | 5118 |
| TNFRSF4 | 1.606404557 | 7293 |
| TMEM102 | 1.132650893 | 284114 |
| AC005046.1 | 1.346133323 | NA |
| TK1 | 1.757317438 | 7083 |
| CHEK2 | 1.202557375 | 11200 |
| LINC01686 | 1.327628699 | 284648 |
| SCARF2 | 1.142810668 | 91179 |
| GINS2 | 1.461424078 | 51659 |
| AC023794.1 | 1.691141659 | NA |
| FGF8 | 1.7071643 | 2253 |
| SERINC2 | 1.794660998 | 347735 |
| FAM20A | 1.849386542 | 54757 |
| AC010913.1 | 1.369390781 | NA |
| GRM4 | 2.053731939 | 2914 |
| HOXC9 | 3.843864063 | 3225 |
| LINC01971 | 1.348608048 | 105371927 |
| DKK2 | 2.306598185 | 27123 |
| HERC2P10 | 1.460019381 | 390561 |
| E2F1 | 1.434699656 | 1869 |
| RPL18A | 1.036112409 | 6142 |
| HIST1H2BG | 1.694321258 | NA |
| CDCA5 | 1.606221448 | 113130 |
| MEGF6 | 1.458064334 | 1953 |
| TNNI1 | 2.317310289 | 7135 |
| HNRNPA1P21 | 1.674436397 | 344697 |
| IGF2BP1 | 2.568739633 | 10642 |
| AL590326.1 | 1.593302674 | NA |
| PPP1R14BP3 | 1.109830584 | 100507617 |
| PARPBP | 1.258371738 | 55010 |
| NKX6-1 | 1.922573785 | 4825 |
| MMP25 | 1.492576899 | 64386 |
| C21orf58 | 1.120439052 | 54058 |
| TMPO-AS1 | 1.09083668 | 100128191 |
| LINC00858 | 2.285797752 | 170425 |
| SLC43A1 | 1.123883082 | 8501 |
| AL445670.1 | 2.025447724 | NA |
| LAMP3 | 1.892039752 | 27074 |
| C14orf39 | 2.493888259 | 317761 |
| FOXE3 | 1.861144389 | 2301 |
| AP000919.4 | 1.896636461 | NA |
| LRRC26 | 2.228837458 | 389816 |
| SMTNL2 | 1.461445556 | 342527 |
| AC021594.2 | 2.131946352 | NA |
| CRIP1 | 1.403979292 | 1396 |
| ZNF695 | 1.517815595 | 57116 |
| CLEC10A | 2.251784192 | 10462 |
| GCSAML | 2.243250735 | 148823 |
| ADAMTS19-AS1 | 1.983565475 | 103689846 |
| PDLIM7 | 1.185263718 | 9260 |
| AC011447.6 | 1.503806798 | NA |
| MIR1915HG | 1.497664239 | 399726 |
| HIST2H2BC | 1.29358143 | NA |
| FUT7 | 1.323189424 | 2529 |
| AL353613.1 | 3.791824282 | NA |
| HOXB-AS3 | 4.059677007 | 404266 |
| GALNT12 | 1.278877724 | 79695 |
| HAPLN3 | 1.364347004 | 145864 |
| FAM242C | 1.951191283 | 112488744 |
| NR5A1 | 1.9795339 | 2516 |
| AURKB | 2.075249957 | 9212 |
| OR4A16 | 4.662560056 | 81327 |
| NRM | 1.137351527 | 11270 |
| CHAF1A | 1.081516341 | 10036 |
| CCNA2 | 1.610681764 | 890 |
| CDC6 | 1.550744194 | 990 |
| FGF3 | 4.438878437 | 2248 |
| LINC02244 | 1.862746645 | 105371018 |
| AC091100.1 | 1.572057651 | NA |
| SP9 | 2.231505236 | 100131390 |
| CDH3 | 1.587711624 | 1001 |
| TIMELESS | 1.191016015 | 8914 |
| TNFRSF18 | 1.682042906 | 8784 |
| CHIT1 | 2.449984594 | 1118 |
| RASSF1-AS1 | 1.078172537 | 102060282 |
| EPOP | 1.12920417 | 100170841 |
| MAP3K21 | 1.693121968 | 84451 |
| CCDC85B | 1.258997462 | 11007 |
| MND1 | 1.53897912 | 84057 |
| TMED10P2 | 1.85774535 | 100129727 |
| LINC01705 | 2.611763346 | 107985307 |
| TEAD2 | 1.37501465 | 8463 |
| ORC6 | 1.288167445 | 23594 |
| UNC5B | 1.030501756 | 219699 |
| C4orf48 | 1.281673874 | 401115 |
| CCNYL2 | 1.968283035 | 414194 |
| AC106786.1 | 2.198674209 | NA |
| MYBL2 | 2.161095923 | 4605 |
| LTB4R | 1.031944684 | 1241 |
| LAMB1 | 1.680908298 | 3912 |
| C1QTNF12 | 1.365586194 | 388581 |
| LINC02084 | 1.997661669 | 105377006 |
| TROAP | 1.969040751 | 10024 |
| HTRA3 | 1.932947776 | 94031 |
| SPAG5 | 1.264782308 | 10615 |
| FANCI | 1.41181979 | 55215 |
| KCNQ1OT1 | 1.210012942 | 10984 |
| FAM220CP | 1.39306839 | 100507415 |
| MCM10 | 1.938714916 | 55388 |
| CHTF18 | 1.125520321 | 63922 |
| AC004585.1 | 2.552377889 | NA |
| AC006206.2 | 3.712279526 | NA |
| MTCO1P53 | 2.358372918 | 107075285 |
| CKS2 | 1.283196959 | 1164 |
| AL035461.2 | 1.179030453 | NA |
| CCNB2 | 1.836079945 | 9133 |
| AC141557.1 | 2.200930223 | NA |
| AKNAD1 | 1.442396792 | 254268 |
| KISS1R | 2.450875357 | 84634 |
| UBE2S | 1.094753561 | 27338 |
| AC008060.2 | 3.20614163 | NA |
| DOK2 | 1.738171232 | 9046 |
| FZD9 | 1.280624598 | 8326 |
| LINC01096 | 3.448136809 | 285548 |
| NID1 | 1.347104806 | 4811 |
| AL354861.3 | 1.637458739 | NA |
| CHRDL2 | 1.879480715 | 25884 |
| Z97205.2 | 1.610005848 | NA |
| CDC25A | 1.24162621 | 993 |
| C17orf53 | 1.056401507 | NA |
| HELLS | 1.269767143 | 3070 |
| SRD5A2 | 2.338579025 | 6716 |
| COL11A2 | 1.186089121 | 1302 |
| HOXB8 | 3.878767128 | 3218 |
| MYB | 1.736671918 | 4602 |
| PTPRU | 1.330562248 | 10076 |
| CDC45 | 1.769388297 | 8318 |
| LTB | 1.288596007 | 4050 |
| HOXC6 | 3.118032432 | 3223 |
| HIST1H1C | 1.390968325 | NA |
| EME1 | 1.302375497 | 146956 |
| AC026403.1 | 1.378713311 | NA |
| TMPRSS2 | 1.847894971 | 7113 |
| NUF2 | 1.589742938 | 83540 |
| AC011458.1 | 1.165836719 | NA |
| AC026333.4 | 1.41305086 | NA |
| AC026401.3 | 1.373985606 | NA |
| LINC01614 | 2.569018583 | 105373869 |
| AC007448.4 | 1.295018595 | NA |
| AC011447.7 | 1.161924204 | NA |
| TPBG | 1.687261463 | 7162 |
| CACNA1G | 1.678381194 | 8913 |
| HIST1H2AM | 1.834714891 | NA |
| LHX1-DT | 2.59049562 | 102723471 |
| MMP15 | 1.028858827 | 4324 |
| SI | 2.409047123 | 6476 |
| DHRS2 | 2.082622934 | 10202 |
| LINC01857 | 2.718373079 | 102724714 |
| MAML3 | 1.062597789 | 55534 |
| ELFN1-AS1 | 1.76190847 | 101927125 |
| LINC00624 | 1.178539945 | 100289211 |
| PCDHB11 | 1.256133612 | 56125 |
| LINC01571 | 2.279345458 | 101927364 |
| AC015813.4 | 1.525261968 | NA |
| PLAGL1 | 1.318348161 | 5325 |
| CCDC78 | 1.280414861 | 124093 |
| LINC01738 | 2.787472497 | 107984953 |
| MTFR2 | 1.285352427 | 113115 |
| EFNA4 | 1.16283463 | 1945 |
| AC090517.2 | 1.025497881 | NA |
| SLC1A3 | -1.372380845 | 6507 |
| XPNPEP2 | 1.371349868 | 7512 |
| LINC02367 | 1.930198011 | 101930452 |
| STK31 | 1.40177327 | 56164 |
| HOXC-AS2 | 3.420864972 | 100874364 |
| GZMM | 1.227572634 | 3004 |
| TCF7 | 1.328056457 | 6932 |
| PCOLCE-AS1 | 1.483454415 | 100129845 |
| TRIP13 | 1.260275446 | 9319 |
| MMP17 | 1.481884487 | 4326 |
| DIO3OS | 1.500217764 | 64150 |
| CCNB1 | 1.315474089 | 891 |
| GPC3 | 2.045432014 | 2719 |
| LOXL1 | 1.882604337 | 4016 |
| ENO3 | 1.030419752 | 2027 |
| UTF1 | 2.205023243 | 8433 |
| C9orf106 | 1.187772318 | 414318 |
| IGF2BP2 | 2.26590841 | 10644 |
| PHLDA2 | 2.047554962 | 7262 |
| F12 | 1.417973033 | 2161 |
| RPL21P28 | 1.182741033 | 100131205 |
| RPL7AP50 | 1.078150143 | 87688 |
| AC116533.1 | 1.014637453 | NA |
| AL139246.5 | 1.891143003 | NA |
| ENTHD1 | 2.183523981 | 150350 |
| EEF1G | 1.171396628 | 1937 |
| AC026774.1 | 2.233267047 | NA |
| ALDH1A3 | 2.407485725 | 220 |
| MNX1-AS2 | 1.864568978 | 105375606 |
| PLK1 | 1.250144935 | 5347 |
| EFCC1 | 1.286037896 | 79825 |
| KDELR3 | 1.45180887 | 11015 |
| AVPR1A | 1.562493158 | 552 |
| LINC01833 | 2.795378168 | 107985879 |
| TMSB10 | 1.069720286 | 9168 |
| AC145098.2 | 1.37980063 | NA |
| BRIP1 | 1.651278994 | 83990 |
| GINS1 | 1.139864637 | 9837 |
| LINC00887 | 1.863317891 | 100131551 |
| CLEC4M | 2.525254672 | 10332 |
| TYMS | 1.448324869 | 7298 |
| CDCA4P4 | -1.452085285 | 100188945 |
| ELOVL3 | 1.676300201 | 83401 |
| AC025627.1 | 1.837090626 | NA |
| ORC1 | 1.523488173 | 4998 |
| LINC01054 | 2.012190599 | 104355150 |
| ATP6V1B1-AS1 | 1.476399496 | 101927750 |
| P2RX2 | 2.554394628 | 22953 |
| PCLAF | 1.677550428 | 9768 |
| AC011474.4 | 1.270636387 | NA |
| EPHB2 | 1.202200358 | 2048 |
| HNRNPA3P3 | 1.153434148 | 643689 |
| CENPH | 1.038151936 | 64946 |
| HIST1H2BK | 1.152054009 | NA |
| MYCNUT | 1.779257498 | 103752554 |
| TMSB15A | 1.744035904 | 11013 |
| EZH2 | 1.217089154 | 2146 |
| LSINCT5 | 1.968204597 | 101234261 |
| DKKL1 | 1.694088353 | 27120 |
| CCDC8 | 1.801289653 | 83987 |
| ANKRD34B | 1.856677339 | 340120 |
| CDC20 | 1.71725652 | 991 |
| LHX1 | 2.424125637 | 3975 |
| OR2A9P | 1.905811976 | 441295 |
| ITGAD | 1.783561882 | 3681 |
| SMIM25 | 1.65715636 | 100506115 |
| PDIA5 | 1.149082259 | 10954 |
| AC103808.6 | 2.464977452 | NA |
| IGLV1-44 | 3.062442417 | 28823 |
| HES5 | 1.747864981 | 388585 |
| CEP55 | 1.686876066 | 55165 |
| RPS28P7 | 1.595923628 | 646195 |
| CLDN14 | 1.826135701 | 23562 |
| DTL | 1.608981826 | 51514 |
| AC087683.2 | 1.080481716 | NA |
| EMILIN2 | 1.680314747 | 84034 |
| AC246787.1 | 1.074687851 | NA |
| ANPEP | 1.122858391 | 290 |
| HEPACAM2 | 1.465225466 | 253012 |
| ALKAL1 | 2.092547053 | 389658 |
| RN7SL192P | 1.347060233 | 106480974 |
| CENPU | 1.57474746 | 79682 |
| ARHGEF39 | 1.073110253 | 84904 |
| AC012618.1 | 1.166679758 | NA |
| CTSV | 1.500803743 | 1515 |
| CHEK1 | 1.021644172 | 1111 |
| PXDN | 1.183390458 | 7837 |
| ITGB1BP2 | 1.09817534 | 26548 |
| PLAG1 | 1.27866917 | 5324 |
| DEPDC1B | 1.503150903 | 55789 |
| RDH8 | 2.702300181 | 50700 |
| POU3F1 | 1.624351212 | 5453 |
| AHCYL2 | -1.064351817 | 23382 |
| HOXB7 | 2.009947169 | 3217 |
| KIF18A | 1.552298159 | 81930 |
| FST | 1.466368692 | 10468 |
| LINC02577 | 3.777743729 | 111216280 |
| AC011450.1 | 1.315817827 | NA |
| FAM83D | 1.533998397 | 81610 |
| PDE10A | 1.152181817 | 10846 |
| FCGR2B | 1.967277586 | 2213 |
| OTOP2 | 2.453282185 | 92736 |
| CENPA | 1.737207382 | 1058 |
| LINC00261 | 3.774244485 | 140828 |
| HIST2H2BD | 1.25009887 | NA |
| COL27A1 | 1.358335338 | 85301 |
| AC012676.4 | 1.441220007 | NA |
| KIF23 | 1.649509949 | 9493 |
| AC007192.1 | 1.425287244 | NA |
| CDK1 | 1.564143093 | 983 |
| NPW | 1.639209568 | 283869 |
| MXRA5 | 1.801301295 | 25878 |
| FRMD1 | 1.938560431 | 79981 |
| SCGB3A1 | 1.499370706 | 92304 |
| EMILIN1 | 1.177503271 | 11117 |
| MBL1P | 1.174908973 | 8512 |
| HIST1H2AE | 1.352121885 | NA |
| TP53I3 | 1.077582029 | 9540 |
| CENPM | 1.286869471 | 79019 |
| AC244090.3 | 1.115622434 | NA |
| HIST1H3A | 1.484325723 | NA |
| C1orf127 | 1.373618287 | 148345 |
| REC8 | 1.007694552 | 9985 |
| LMNB1 | 1.193123106 | 4001 |
| DIO3 | 1.637920704 | 1735 |
| SVIL2P | 1.297513558 | 645954 |
| TIFAB | 1.870127501 | 497189 |
| HK3 | 1.362862479 | 3101 |
| BIRC5 | 1.76925931 | 332 |
| AC130371.2 | 1.087985348 | NA |
| PDLIM5 | -1.040143014 | 10611 |
| BHLHE22 | 1.971613977 | 27319 |
| SVIL | 1.105626114 | 6840 |
| EFNA3 | 1.023625161 | 1944 |
| TRIM71 | 1.856171472 | 131405 |
| AL355916.2 | -2.989531777 | NA |
| UBE2C | 1.789039091 | 11065 |
| RPL13AP25 | 1.021150002 | 100287887 |
| POLE2 | 1.014610418 | 5427 |
| WDR62 | 1.438565802 | 284403 |
| JAML | 1.681128621 | 120425 |
| AC105118.1 | 2.599108706 | NA |
| AC092685.1 | 2.422642177 | NA |
| AC008608.2 | 1.009722719 | NA |
| MARCO | 2.210369974 | 8685 |
| BMP6 | 1.259559637 | 654 |
| XDH | 1.86294277 | 7498 |
| SGO1 | 1.60275457 | 151648 |
| AC007250.1 | 1.656587757 | NA |
| RASSF10 | 2.140496993 | 644943 |
| TM4SF19 | 1.448787092 | 116211 |
| HOXC13 | 3.585632654 | 3229 |
| AL353597.3 | 1.867085309 | NA |
| OIP5 | 1.256142448 | 11339 |
| TACC3 | 1.397090905 | 10460 |
| IGHV3-48 | 3.583492189 | 28424 |
| EOMES | 1.788732365 | 8320 |
| ARHGEF26-AS1 | -1.14358504 | 100507524 |
| GHRHR | -1.640504085 | 2692 |
| SULT1C4 | -1.225784777 | 27233 |
| AC006329.2 | 1.355913987 | NA |
| AL449043.1 | 1.675676312 | NA |
| AC009054.2 | 1.141959659 | NA |
| NRTN | 1.922416018 | 4902 |
| MYCN | 1.136614683 | 4613 |
| LINC01033 | 1.996571684 | 104355136 |
| SPARC | -1.542361885 | 6678 |
| IGFL2 | 1.538531455 | 147920 |
| GP2 | 2.799308103 | 2813 |
| RAD51AP1 | 1.290207311 | 10635 |
| AL161908.1 | 2.388103399 | NA |
| AC092718.4 | 1.022225461 | NA |
| PERM1 | 1.337311955 | 84808 |
| SIX5 | 1.105011006 | 147912 |
| CHRNA5 | 1.089668043 | 1138 |
| MASP1 | -1.414012391 | 5648 |
| WNT4 | 1.529559831 | 54361 |
| ZWINT | 1.159408504 | 11130 |
| FANCA | 1.072794978 | 2175 |
| IHH | 1.349901099 | 3549 |
| TBL1XR1-AS1 | 1.783902576 | 100874217 |
| SUCNR1 | 1.427520221 | 56670 |
| AC090774.2 | 2.185998494 | NA |
| DCSTAMP | 2.000953802 | 81501 |
| DTNA | -1.031946958 | 1837 |
| ADIRF-AS1 | 1.265532341 | 100133190 |
| RMI2 | 1.247697225 | 116028 |
| AL645608.8 | 1.67218083 | NA |
| AC024884.2 | 1.857528376 | NA |
| MIR7152 | 1.74606925 | 102465689 |
| HUNK | 1.253961842 | 30811 |
| AC005224.3 | 1.830209433 | NA |
| CDKN3 | 1.243260707 | 1033 |
| CRYM-AS1 | 1.352280013 | 400508 |
| TUBB6 | 1.342987963 | 84617 |
| AC007952.4 | 1.77014261 | NA |
| TRBV28 | 1.860841273 | 28559 |
| MCAM | 1.046310188 | 4162 |
| KIF11 | 1.19145301 | 3832 |
| AC011442.1 | 1.218794159 | NA |
| DNAJC5B | 1.427501316 | 85479 |
| ANO9 | 1.528449256 | 338440 |
| LINC02487 | 1.4132143 | 441178 |
| AC079684.1 | 1.139518097 | NA |
| AC027287.2 | 2.373937501 | NA |
| NEUROG2 | 1.888442464 | 63973 |
| SKA3 | 1.445049981 | 221150 |
| ESPL1 | 1.583803724 | 9700 |
| CDCA2 | 1.553200114 | 157313 |
| DLGAP5 | 1.788855323 | 9787 |
| FGF18 | 1.420462389 | 8817 |
| PIK3CD-AS2 | 1.546192178 | 101929074 |
| CCL26 | 1.491501344 | 10344 |
| CTHRC1 | 1.654760587 | 115908 |
| KIF2C | 1.501815178 | 11004 |
| ASF1B | 1.486387212 | 55723 |
| ATP2A1 | 1.106723752 | 487 |
| LOXL2 | 1.34732193 | 4017 |
| BMP3 | 1.859754269 | 651 |
| DLX4 | 1.432401322 | 1748 |
| EVA1B | 1.016073076 | 55194 |
| AC027228.2 | 1.338053814 | NA |
| GAPLINC | 1.770104208 | 100505592 |
| CCDC3 | 1.252795932 | 83643 |
| DAO | -4.663580282 | 1610 |
| AC007040.2 | 1.036819417 | NA |
| MICE | 1.468138688 | 4280 |
| AC007497.1 | 1.092048775 | NA |
| SLAMF1 | 1.858606638 | 6504 |
| NDST1-AS1 | 1.168572165 | 102546298 |
| AL355803.1 | 1.386521349 | NA |
| MRO | -1.531948792 | 83876 |
| TWIST1 | 1.441737837 | 7291 |
| RPL13P12 | 1.094491847 | 388344 |
| VAX2 | 1.155276938 | 25806 |
| CCDC150 | 1.033291731 | 284992 |
| THEGL | 1.629723075 | 100506564 |
| SYNPO2L | 1.380234452 | 79933 |
| AC012676.3 | 1.139021258 | NA |
| GAPDHP70 | 1.379136714 | 642259 |
| AC106820.2 | 1.051677457 | NA |
| KIF4A | 1.444887343 | 24137 |
| ANKRD18CP | 1.279327125 | 101926917 |
| AL133325.3 | 1.139698287 | NA |
| CLMP | 1.211276346 | 79827 |
| SLC47A1 | 1.423905157 | 55244 |
| HAND1 | 2.305297544 | 9421 |
| E2F2 | 1.584206385 | 1870 |
| AUNIP | 1.020520449 | 79000 |
| OSR2 | 2.251337609 | 116039 |
| LOXL1-AS1 | 1.666823383 | 100287616 |
| AC108134.1 | 1.195177295 | NA |
| CRYBA4 | 1.404668766 | 1413 |
| LUM | 1.690820502 | 4060 |
| PRSS1 | 3.002447365 | 5644 |
| COL6A3 | 1.927289057 | 1293 |
| COL4A2 | 1.554220066 | 1284 |
| FBXL21P | 1.844502228 | 26223 |
| LINC01116 | 1.718527051 | 375295 |
| HASPIN | 1.42859058 | 83903 |
| PLAT | 1.605689254 | 5327 |
| AC112487.1 | 1.755128242 | NA |
| SNRPFP2 | 1.271935815 | 100874411 |
| XKR5 | 1.529205351 | 389610 |
| NKX2-1 | 2.033113755 | 7080 |
| HTRA4 | 1.894299417 | 203100 |
| FAM167B | 1.012372873 | 84734 |
| CCKBR | 1.899267036 | 887 |
| RNF212 | 1.469773587 | 285498 |
| HIST3H2BA | 2.02227335 | NA |
| CDC25C | 1.450415639 | 995 |
| PTGIR | 1.113933903 | 5739 |
| AC091138.1 | 1.349360293 | NA |
| MMP2 | 1.094960967 | 4313 |
| HIST1H3J | 1.677441646 | NA |
| IGHA2 | 2.636959435 | 3494 |
| ALDH1L1 | -1.554603542 | 10840 |
| HCAR1 | 1.367974016 | 27198 |
| MCIDAS | 2.257383919 | 345643 |
| PRC1 | 1.108171466 | 9055 |
| XRCC2 | 1.122066781 | 7516 |
| WNT9A | 1.333867872 | 7483 |
| COL10A1 | 1.473047798 | 1300 |
| AC078850.1 | 1.706761923 | NA |
| GAS2L3 | 1.429961677 | 283431 |
| ZBED2 | 2.08869728 | 79413 |
| CD68 | 1.06400369 | 968 |
| RPL34-AS1 | -1.012005506 | 285456 |
| ACTBP12 | 2.538365243 | 100462767 |
| AMN | 1.044022619 | 81693 |
| MNX1-AS1 | 2.177418083 | 645249 |
| ANXA2P3 | 1.727452794 | 305 |
| IL2RG | 1.24453152 | 3561 |
| KCNG2 | 1.262655569 | 26251 |
| NWD2 | 2.049133071 | 57495 |
| LHFPL1 | -1.768621434 | 340596 |
| HOXD9 | 2.464357886 | 3235 |
| LGALS7B | 2.483269404 | 653499 |
| EPHA7 | 1.526615113 | 2045 |
| KIFC1 | 1.446729898 | 3833 |
| DNAH3 | 1.878929199 | 55567 |
| AL121906.1 | 1.383779585 | NA |
| LINC00337 | 1.396805371 | 148645 |
| BLOC1S5-TXNDC5 | 1.183683326 | 100526836 |
| RNF43 | 1.481311728 | 54894 |
| SV2C | 1.658025852 | 22987 |
| BUB1 | 1.395457557 | 699 |
| ARHGAP11A | 1.170340305 | 9824 |
| A2ML1 | -1.741837041 | 144568 |
| HOXC-AS1 | 3.179336609 | 100874363 |
| ZNF492 | 1.288745365 | 57615 |
| CDH15 | 1.778872966 | 1013 |
| TPX2 | 1.361849088 | 22974 |
| WNT5A-AS1 | 1.188585104 | 100874008 |
| RPSAP7 | 1.249875568 | 654506 |
| CHAF1B | 1.164767598 | 8208 |
| NECTIN4 | 1.283839132 | 81607 |
| RAD54L | 1.249171804 | 8438 |
| CYP4F29P | 1.918618853 | 54055 |
| NARF-AS1 | 1.239302638 | 111082991 |
| LINC01558 | 1.380283704 | 26238 |
| ZNF887P | 1.200467778 | 100419709 |
| NCAPH | 1.321723457 | 23397 |
| AC233992.3 | 1.093500562 | NA |
| TRPC6 | 1.158910619 | 7225 |
| ZAP70 | 1.17128692 | 7535 |
| MEST | 1.052319513 | 4232 |
| AC107954.1 | 1.47834823 | NA |
| HES4 | 1.144666385 | 57801 |
| AC113410.3 | 1.511583065 | NA |
| NDC80 | 1.48494492 | 10403 |
| ZYG11A | 1.781787948 | 440590 |
| SYTL4 | -1.464462644 | 94121 |
| AC018410.2 | 1.286629136 | NA |
| LINC01905 | 1.482960641 | 102724698 |
| AC021016.3 | 1.331805341 | NA |
| ANKRD20A19P | 1.852454874 | 400110 |
| DNAJC5G | 1.706848936 | 285126 |
| COL26A1 | 1.381194125 | 136227 |
| SLCO4A1-AS1 | 1.60344796 | 100127888 |
| GJC3 | 1.200344172 | 349149 |
| LINC01482 | 1.59511526 | 101928104 |
| SIAH3 | 1.662842209 | 283514 |
| EFNA2 | 1.028367755 | 1943 |
| CHRNA3 | 1.567500056 | 1136 |
| AC004057.1 | 1.39123348 | NA |
| YBX2 | 1.261435444 | 51087 |
| CACNG6 | 1.211059975 | 59285 |
| MSH4 | 1.12499146 | 4438 |
| AC087473.1 | 1.668923977 | NA |
| CCR7 | 1.43343409 | 1236 |
| HAND2-AS1 | 2.476658248 | 79804 |
| FAM72C | 1.128458407 | 554282 |
| AL031777.1 | 1.282847823 | NA |
| POMC | 1.00052712 | 5443 |
| NFE4 | 1.54045507 | 58160 |
| ANKRD26P3 | 1.783283738 | 100101938 |
| PLK4 | 1.010663555 | 10733 |
| AC022211.3 | 1.043137659 | NA |
| PIF1 | 1.188026325 | 80119 |
| TFPI2 | 1.688620064 | 7980 |
| COL28A1 | -2.608262182 | 340267 |
| AL135786.1 | -1.173205329 | NA |
| KIF14 | 1.432325491 | 9928 |
| SIM2 | 1.067507033 | 6493 |
| TDO2 | 1.37369191 | 6999 |
| ECSCR | 1.1294636 | 641700 |
| KLRD1 | 1.004860504 | 3824 |
| KRT1 | 2.01674191 | 3848 |
| AP000424.1 | -1.922643055 | NA |
| NR5A2 | 1.422569689 | 2494 |
| AC025754.2 | 1.054297762 | NA |
| C17orf82 | 1.109630644 | 388407 |
| TNFRSF9 | 1.354913879 | 3604 |
| POLQ | 1.302882329 | 10721 |
| LIX1 | -1.516065912 | 167410 |
| MAOA | -1.218907192 | 4128 |
| PIEZO2 | 1.283438117 | 63895 |
| SLC14A2 | -2.775385841 | 8170 |
| AC026780.1 | -2.058074033 | NA |
| HMX1 | 1.29933259 | 3166 |
| DTHD1 | -2.639954428 | 401124 |
| GJC1 | 1.07456511 | 10052 |
| RTN4RL1 | 1.6141563 | 146760 |
| PON2 | -1.007839774 | 5445 |
| AC079140.2 | 1.101315492 | NA |
| CDCA8 | 1.325376446 | 55143 |
| NEK2 | 1.446095826 | 4751 |
| AURKA | 1.031253731 | 6790 |
| NUSAP1 | 1.368508633 | 51203 |
| ERCC6L | 1.252244429 | 54821 |
| AC007240.1 | 2.114002823 | NA |
| HMMR | 1.24809272 | 3161 |
| PSTPIP2 | 1.003825418 | 9050 |
| HCCAT5 | 1.513400247 | 283902 |
| SIGLEC17P | 1.256679216 | 284367 |
| LINC01565 | 1.469247141 | 23434 |
| AC022211.1 | 1.125943523 | NA |
| CPA3 | 2.539048107 | 1359 |
| MCOLN2 | 1.465646333 | 255231 |
| HOXD11 | 3.05899174 | 3237 |
| AC007998.3 | 1.258486729 | NA |
| USH1C | -2.326641938 | 10083 |
| FABP6 | 1.584232941 | 2172 |
| DLX2 | 1.768268807 | 1746 |
| FGF9 | 1.213046611 | 2254 |
| FANCD2OS | 1.48055656 | 115795 |
| CENPK | 1.300859521 | 64105 |
| AC006450.2 | 1.252480073 | NA |
| AC254629.1 | 1.62147387 | NA |
| LDLRAD4-AS1 | -1.895138485 | 100288122 |
| TRABD2A | 1.307539281 | 129293 |
| PRDM16-DT | 1.26021286 | 440556 |
| TNNI3 | 1.310905119 | 7137 |
| SNX18P3 | 1.438921838 | 100418975 |
| AC087481.2 | 1.873479434 | NA |
| AL121894.1 | -1.716638186 | NA |
| AC009121.2 | 1.539848994 | NA |
| HIST1H3D | 1.095086246 | NA |
| HOXB3 | 2.219972776 | 3213 |
| PKMYT1 | 1.117383662 | 9088 |
| AP006621.1 | 1.143336032 | NA |
| NWD1 | -2.128774533 | 284434 |
| AC087481.1 | 1.444868615 | NA |
| PLVAP | 1.099208817 | 83483 |
| FRAS1 | 1.310929292 | 80144 |
| AP000553.2 | 1.086530083 | NA |
| PRSS30P | 1.456142024 | 124221 |
| FAM9C | 1.718967614 | 171484 |
| AC023421.1 | -2.784587354 | NA |
| ANGPTL1 | -1.852099882 | 9068 |
| OR7E29P | -1.385887136 | 26644 |
| AC011446.1 | 1.345788465 | NA |
| CDRT15P1 | 1.052282999 | 94158 |
| CCER2 | 1.364466496 | 643669 |
| GREM1 | 1.652202369 | 26585 |
| HIST2H4A | 1.16081091 | NA |
| CES1 | 1.610849526 | 1066 |
| LINC02370 | 1.757166902 | 338797 |
| BCO1 | -1.362761507 | 53630 |
| SERPINH1 | 1.089601355 | 871 |
| AP003392.3 | 1.103558545 | NA |
| LINC01436 | 2.476452573 | 100996609 |
| EXO1 | 1.409289131 | 9156 |
| GPR75 | -1.180420666 | 10936 |
| ISM2 | 1.332356698 | 145501 |
| ARHGEF35 | 1.367490792 | 445328 |
| LINC01322 | -1.694396752 | 103695433 |
| NPY5R | 1.655809535 | 4889 |
| AP001324.3 | 1.403206001 | NA |
| KCNG1 | 1.601314569 | 3755 |
| FSTL1 | 1.110437032 | 11167 |
| MIR3186 | 1.094086641 | 100422944 |
| LINC01524 | 2.198046126 | 101927700 |
| TMC1 | -1.381620498 | 117531 |
| AC107886.1 | 1.824972134 | NA |
| PIMREG | 1.475066961 | 54478 |
| DBX2 | -1.870370257 | 440097 |
| CD5L | 1.836514915 | 922 |
| ESCO2 | 1.382689529 | 157570 |
| AC025062.1 | 1.158470203 | NA |
| UCN2 | 1.600457737 | 90226 |
| SPARCL1 | -1.014429971 | 8404 |
| HCN4 | 1.407457755 | 10021 |
| HSPG2 | 1.26452109 | 3339 |
| AC046168.2 | 1.258273833 | NA |
| SNAI2 | 1.164603664 | 6591 |
| NKX2-4 | 2.228878515 | 644524 |
| DSC3 | 2.514810973 | 1825 |
| AC011504.1 | 1.199748185 | NA |
| ASPM | 1.548273216 | 259266 |
| SKA1 | 1.34628227 | 220134 |
| ESM1 | 2.004148169 | 11082 |
| AC116351.2 | 1.343114445 | NA |
| AC004080.15 | 2.525868834 | NA |
| LINC01960 | 1.482247712 | 100128905 |
| AL157400.2 | 1.434159969 | NA |
| MYH2 | 2.148460465 | 4620 |
| HJURP | 1.445954548 | 55355 |
| FOXCUT | 1.221888259 | 101927703 |
| BARX2 | 1.469616518 | 8538 |
| AC113143.1 | 1.259805487 | NA |
| ADCYAP1R1 | -1.223524508 | 117 |
| NPAS1 | 1.154460625 | 4861 |
| DDX11L5 | 2.671116487 | 100287596 |
| AC002075.2 | 1.004275528 | NA |
| HCG20 | -1.304261583 | 105375013 |
| ONECUT2 | 1.0399612 | 9480 |
| NCAPG | 1.416883625 | 64151 |
| KRT17P4 | 1.56927032 | 339186 |
| AL358473.1 | 1.15913903 | NA |
| MIR1-1HG-AS1 | 1.8150455 | 253868 |
| RTKN2 | 1.083560139 | 219790 |
| CPNE5 | -1.322101414 | 57699 |
| THSD7B | 1.025710637 | 80731 |
| AC126564.1 | -3.285765442 | NA |
| AC010336.2 | 1.032116385 | NA |
| AC124854.1 | -1.550000687 | NA |
| ZFPM2-AS1 | 1.232229468 | 102723356 |
| LINC00664 | 1.055333228 | 400680 |
| IL12B | 1.664811789 | 3593 |
| SOX11 | 1.024223092 | 6664 |
| MELK | 1.426250356 | 9833 |
| PCAT7 | 1.754995418 | 101928099 |
| DQX1 | 1.089202516 | 165545 |
| AC108025.1 | 1.46085923 | NA |
| EVPL | 1.515609805 | 2125 |
| GLI1 | 1.65386363 | 2735 |
| AC019068.1 | 1.252715505 | NA |
| AC063926.2 | 1.712055702 | NA |
| FIBCD1 | 1.206120246 | 84929 |
| AC079298.3 | -1.921874494 | NA |
| UNCX | 2.89804076 | 340260 |
| HOXC8 | 2.712803993 | 3224 |
| AC079584.1 | -3.139141789 | NA |
| KCNG3 | 1.798457196 | 170850 |
| HOTAIR | 3.097906259 | 100124700 |
| TTK | 1.354673847 | 7272 |
| KLK2 | 2.731909186 | 3817 |
| KIF15 | 1.005122183 | 56992 |
| PLEKHN1 | 1.100861658 | 84069 |
| MMP7 | 1.993862609 | 4316 |
| BNIP3P28 | 1.712326419 | 106481966 |
| AL139022.2 | -1.056822784 | NA |
| RADX | -1.199230664 | 55086 |
| OVOL3 | 1.184104879 | 728361 |
| KLK4 | 1.836798438 | 9622 |
| FAM187B | 1.434970958 | 148109 |
| TRPC5OS | -1.481771896 | 100329135 |
| AC005225.4 | -1.06657811 | NA |
| AC063979.2 | -1.466515778 | NA |
| PBK | 1.414640714 | 55872 |
| SHCBP1 | 1.077851701 | 79801 |
| AC037471.1 | -4.288171537 | NA |
| CETP | 1.06268384 | 1071 |
| GPR50-AS1 | 2.122794751 | 100128688 |
| GPX8 | 1.518849067 | 493869 |
| HSPD1P6 | 1.258645636 | 645548 |
| CXCL5 | 1.325133385 | 6374 |
| CD300LG | -2.320772627 | 146894 |
| AL136084.3 | 2.153926317 | NA |
| CLSPN | 1.267497247 | 63967 |
| HOXC10 | 2.697944559 | 3226 |
| USH1G | 1.369530996 | 124590 |
| MAPK15 | 1.799263639 | 225689 |
| LINC00994 | -1.801546645 | 100287879 |
| TSPEAR-AS2 | 1.175130708 | 114043 |
| CENPF | 1.328303379 | 1063 |
| AC073263.1 | 1.29141895 | NA |
| SNRPGP4 | 1.561460257 | 100130368 |
| AC023449.2 | -1.025294693 | NA |
| SLC6A11 | -2.075774087 | 6538 |
| FAM47E-STBD1 | -1.062475745 | 100631383 |
| LGR6 | -3.876118362 | 59352 |
| GATA3 | 1.475552762 | 2625 |
| MS4A2 | 2.419162685 | 2206 |
| AC023886.1 | 1.695487054 | NA |
| AC004112.1 | -1.059787824 | NA |
| COL4A1 | 1.445090459 | 1282 |
| HIST1H1B | 1.684754641 | NA |
| KCNJ16 | -1.776909359 | 3773 |
| DACH1 | 1.066098899 | 1602 |
| CXorf65 | 1.278382644 | 158830 |
| LINC02234 | -1.507953307 | 109729164 |
| KIF20A | 1.397552025 | 10112 |
| MXRA5Y | 1.776425718 | 286544 |
| AC091874.1 | -2.499347463 | NA |
| DEPDC1 | 1.437546673 | 55635 |
| AF233439.1 | 1.293486326 | NA |
| C2orf73 | -1.144691103 | 129852 |
| RET | 1.286469407 | 5979 |
| AC005999.1 | -1.962532713 | NA |
| FBLIM1 | 1.086823296 | 54751 |
| FOXA1 | 2.01385828 | 3169 |
| GRIK1-AS1 | -1.21812572 | 642976 |
| ATP6V0D2 | 1.80832986 | 245972 |
| AC002454.1 | 1.898095461 | NA |
| RN7SL320P | -1.370919909 | 106481010 |
| LINC01690 | -1.134973464 | 102724502 |
| AC009093.1 | 1.283153681 | NA |
| SIGLEC6 | 1.89669234 | 946 |
| RPL35P5 | 1.143220463 | 441246 |
| CABP7 | 1.268524044 | 164633 |
| RPL21P42 | -1.296171165 | 100271162 |
| HIF3A | -1.771887557 | 64344 |
| CNN2 | 1.064815851 | 1265 |
| RRM2 | 1.42198811 | 6241 |
| CASQ1 | -1.069153839 | 844 |
| SH2D7 | -1.293914846 | 646892 |
| SIRPB3P | -1.622534258 | 105369219 |
| CCT7P2 | -2.139218719 | 100288772 |
| AL136979.1 | -1.380765968 | NA |
| TTR | -11.74463755 | 7276 |
| HOXD10 | 2.409530559 | 3236 |
| AC117500.5 | 1.354910499 | NA |
| PMEL | 1.164388599 | 6490 |
| SLAMF7 | 1.488672193 | 57823 |
| P2RX3 | 1.145085446 | 5024 |
| AL161668.4 | -1.023018628 | NA |
| SPACA3 | 1.469178798 | 124912 |
| TRGJP2 | -2.311263485 | 6972 |
| EGFR | -1.830672434 | 1956 |
| KCTD14 | -1.12621535 | 65987 |
| AL135786.2 | -1.072277026 | NA |
| SERPINB2 | 2.377387763 | 5055 |
| EVX1 | 1.757342966 | 2128 |
| RUNX3 | 1.018538639 | 864 |
| THSD7A | -1.066646136 | 221981 |
| GFRA2 | 1.254407712 | 2675 |
| HOTTIP | 2.110761951 | 100316868 |
| C6orf132 | 1.115089225 | 647024 |
| PDZD2 | -1.177379485 | 23037 |
| FTCD | 1.087633791 | 10841 |
| AC008525.1 | -1.980520753 | NA |
| SYTL1 | 1.051917532 | 84958 |
| NMBR | -1.136020716 | 4829 |
| AC064847.1 | 1.564382597 | NA |
| TAAR3P | -1.709832285 | 9288 |
| AC092821.1 | 1.28478592 | NA |
| OR51E2 | 1.773113719 | 81285 |
| AL139099.3 | 1.088649392 | NA |
| CHST8 | 1.287990978 | 64377 |
| CCDC144NL | 1.583265991 | 339184 |
| AL365226.1 | 1.043205652 | NA |
| CD3D | 1.48651676 | 915 |
| LHX9 | 1.729938153 | 56956 |
| LCK | 1.232662746 | 3932 |
| VGF | 1.672521614 | 7425 |
| FOXD1 | 1.176889336 | 2297 |
| OR6E1P | -1.051137257 | 79323 |
| AC111182.1 | 1.059210028 | NA |
| PON3 | -1.189147639 | 5446 |
| AC104534.1 | 1.007279391 | NA |
| SLC51B | -1.443650424 | 123264 |
| AL353803.5 | 1.306538265 | NA |
| CKAP2L | 1.330953195 | 150468 |
| PRSS54 | -1.126514987 | 221191 |
| OR3A1 | 1.491212952 | 4994 |
| NKX2-1-AS1 | 2.070135281 | 100506237 |
| TMEM252 | -1.924641713 | 169693 |
| AC112236.1 | -2.782075648 | NA |
| AC135050.2 | 1.107133874 | NA |
| PRDM12 | 1.2472401 | 59335 |
| HOXD-AS2 | 1.742603232 | 100506783 |
| ALKAL2 | 1.124473105 | 285016 |
| KNL1 | 1.261428103 | 57082 |
| AC112512.1 | 1.173524977 | NA |
| ROR1-AS1 | 1.763361443 | 101927034 |
| PHKA1P1 | -1.104312354 | 646780 |
| ACRV1 | 1.05859204 | 56 |
| LINC00606 | -1.755196614 | 285370 |
| CLMAT3 | -1.273967454 | 101927096 |
| ATP1A2 | -1.34992003 | 477 |
| AL023803.1 | 1.067025201 | NA |
| LINC00298 | -1.162601501 | 339788 |
| AC015660.2 | 1.225359124 | NA |
| AC020663.3 | 1.006711748 | NA |
| TMEM89 | 1.131412539 | 440955 |
| AJ011932.1 | 1.693101582 | NA |
| LINC01485 | -1.924203657 | 101928154 |
| AC010643.1 | 1.087617721 | NA |
| SLC44A4 | 1.199414307 | 80736 |
| AC006270.1 | 1.097783957 | NA |
| LAD1 | 1.749392303 | 3898 |
| Z94721.1 | 1.073248981 | NA |
| AC011726.1 | -1.536998597 | NA |
| HOXC5 | 2.278450697 | 3222 |
| FRMPD2 | -1.533091723 | 143162 |
| RPS4XP23 | 1.392254483 | 106480153 |
| XCR1 | 1.764235816 | 2829 |
| AL160314.1 | -1.596158727 | NA |
| PTPRB | -1.466880742 | 5787 |
| PPP1R27 | 1.131353943 | 116729 |
| CDKN2C | 1.046651165 | 1031 |
| SNORD60 | 1.059451995 | 26788 |
| AC013270.1 | 1.167184416 | NA |
| AC079140.5 | 1.251995774 | NA |
| CCL5 | 1.263984327 | 6352 |
| AC244153.1 | 1.104818483 | NA |
| AC109635.4 | 2.160346223 | NA |
| LY6G6C | 1.06168015 | 80740 |
| TFCP2L1 | -2.533757525 | 29842 |
| AL023803.2 | 1.408985285 | NA |
| TMEM72 | -3.773023834 | 643236 |
| PNLIP | 2.542010991 | 5406 |
| AC137834.2 | 1.134526255 | NA |
| AL732314.4 | 1.080872538 | NA |
| LINC02251 | -1.57786761 | 105371008 |
| FMO2 | -1.501248447 | 2327 |
| LINC00163 | 1.291185417 | 727699 |
| TLX1 | 1.765205583 | 3195 |
| AL591686.2 | -1.938276751 | NA |
| IDH2-DT | -1.31019225 | 105370966 |
| HAND2 | 2.146680829 | 9464 |
| CST1 | 2.481442586 | 1469 |
| CENPE | 1.178804372 | 1062 |
| FCN1 | 1.35389682 | 2219 |
| CASQ2 | -1.478820041 | 845 |
| HIF1A-AS1 | -1.005039435 | 100750246 |
| THRB-IT1 | 1.492213199 | 100874274 |
| ADRB3 | 1.659092928 | 155 |
| LSP1 | 1.166533209 | 4046 |
| DNMBP-AS1 | -1.781519503 | 100188954 |
| AC008427.1 | 1.169843364 | NA |
| GRAMD4P7 | -3.731052048 | 100631251 |
| ACSS3 | -1.433562831 | 79611 |
| FGFBP2 | -2.117616756 | 83888 |
| CTLA4 | 1.172788743 | 1493 |
| ART1 | -1.6447936 | 417 |
| AC113189.4 | 1.024182164 | NA |
| AC007193.2 | -2.008301571 | NA |
| MEOX2 | -3.724538297 | 4223 |
| MAP3K19 | -2.99819808 | 80122 |
| LINC01929 | 1.73631841 | 101927229 |
| AP005230.1 | 1.763549347 | NA |
| TRDN | -1.912525175 | 10345 |
| EGFR-AS1 | -1.697998181 | 100507500 |
| ITPRID1 | -4.766602083 | 223075 |
| AC004923.1 | 1.222402885 | NA |
| LINC00896 | 1.444097327 | 150197 |
| GABRB1 | -1.366701332 | 2560 |
| COL5A2 | 1.160589788 | 1290 |
| ADAMTS9-AS2 | -1.18436615 | 100507098 |
| AC087477.2 | 1.41838051 | NA |
| MIR210 | 1.422463285 | 406992 |
| ZDBF2 | -1.151279123 | 57683 |
| KIF6 | -1.517616127 | 221458 |
| OSR1 | 1.43450718 | 130497 |
| AP006248.3 | -2.487643822 | NA |
| SLC4A4 | -1.000920447 | 8671 |
| C9orf92 | -2.256963774 | 100129385 |
| AC010425.1 | -1.303394313 | NA |
| RTN1 | -1.012701461 | 6252 |
| AC134312.5 | 1.27593127 | NA |
| STAC | -2.921662033 | 6769 |
| EFCAB9 | 1.160130059 | 285588 |
| AC004899.1 | -2.072566991 | NA |
| UFL1-AS1 | -1.071818671 | 100861530 |
| LEF1-AS1 | 1.045389847 | 641518 |
| AC005487.1 | -2.541423745 | NA |
| AC092112.1 | -1.619807391 | NA |
| B3GAT2 | -1.353668408 | 135152 |
| MIR4520-1 | 1.004684358 | 100616401 |
| ZNF723 | 1.954371724 | 646864 |
| ANGPT1 | -1.65805983 | 284 |
| AC133785.1 | -1.189837003 | NA |
| AC109492.1 | 1.620844498 | NA |
| AL732414.1 | 1.009545304 | NA |
| AC012486.1 | 1.019740333 | NA |
| AC037487.3 | 1.126891518 | NA |
| AC002546.1 | -2.044565537 | NA |
| OR2B6 | 1.199360591 | 26212 |
| LINC01080 | -1.145139355 | 101515984 |
| AC007938.1 | -1.024520187 | NA |
| AQP4 | -1.3089481 | 361 |
| ST8SIA2 | 1.30021881 | 8128 |
| MYLK3 | -1.402056741 | 91807 |
| AHNAK2 | 1.191691655 | 113146 |
| PSD2 | -1.042529146 | 84249 |
| PPP1R14D | -1.178719154 | 54866 |
| MIOX | 1.555689484 | 55586 |
| AL355974.1 | -1.930850655 | NA |
| GLP1R | -2.595604941 | 2740 |
| IRX5 | 1.606855442 | 10265 |
| VIT | -2.096157205 | 5212 |
| AC009878.1 | 1.044236587 | NA |
| AL121956.4 | -1.48850074 | NA |
| RGS13 | 1.301083359 | 6003 |
| SEPTIN14 | -7.101814701 | 346288 |
| AC092675.1 | -1.235255358 | NA |
| LINC02332 | 1.350431276 | 105370400 |
| GRIN3B | 1.051546371 | 116444 |
| GATA5 | 1.684599661 | 140628 |
| AC079777.1 | 1.199315113 | NA |
| SLAMF8 | 1.143536629 | 56833 |
| ATP1A4 | -1.077978843 | 480 |
| AC009884.2 | -1.769691285 | NA |
| AC010975.1 | -1.097196899 | NA |
| CD8B2 | -1.365549263 | 927 |
| AC093278.1 | -1.24434694 | NA |
| AC097065.1 | -1.80279832 | NA |
| HCAR2 | 1.380330617 | 338442 |
| AC083805.3 | 1.249110971 | NA |
| HOXA11 | 2.259412404 | 3207 |
| LINC02355 | 1.137630879 | 101927849 |
| GTSE1 | 1.144406259 | 51512 |
| HSD17B6 | -1.26612062 | 8630 |
| CDK15 | -1.550054512 | 65061 |
| ALPK2 | 1.414645944 | 115701 |
| AP000755.2 | 1.591293973 | NA |
| DLX5 | 1.362856941 | 1749 |
| ANKRD18A | 1.098623311 | 253650 |
| RDH10 | 1.025196505 | 157506 |
| INSYN2B | -1.469066608 | 100131897 |
| ACE | 1.004419106 | 1636 |
| NKX2-5 | 1.964305167 | 1482 |
| AL445426.1 | -1.550136118 | NA |
| AKR1B15 | -1.633028778 | 441282 |
| LINC02620 | 1.622965356 | 101927523 |
| LINC02282 | -1.863116203 | 105370424 |
| AL591686.1 | -1.766342066 | NA |
| NYAP2 | -1.95640476 | 57624 |
| AC007014.2 | 1.153286394 | NA |
| AC073621.1 | -1.510724184 | NA |
| LINC00886 | -1.237118075 | 730091 |
| LINC01235 | -1.860555644 | 401492 |
| EYA1 | -1.303450706 | 2138 |
| AC004899.2 | -1.838552588 | NA |
| ALDH1L1-AS2 | -1.69974022 | 100862662 |
| ISG15 | 1.25987349 | 9636 |
| OTOG | -3.283885619 | 340990 |
| AL033519.1 | -2.093719381 | NA |
| OPRD1 | 1.214030361 | 4985 |
| TMEM196 | -2.006379338 | 256130 |
| AL109809.2 | -1.49612825 | NA |
| L1TD1 | 1.222254069 | 54596 |
| AC007326.1 | -1.263253268 | NA |
| IRF6 | -1.45442889 | 3664 |
| HERC2P5 | 1.390403923 | 388254 |
| AL159174.1 | -1.036404976 | NA |
| AC007128.1 | -1.738976875 | NA |
| AC055876.2 | -1.213651533 | NA |
| PIK3C2G | -1.950841181 | 5288 |
| NOS1 | 1.178578519 | 4842 |
| AC117500.1 | 1.380994316 | NA |
| ABCC6P2 | -1.014205509 | 730013 |
| AC096537.1 | 1.34055743 | NA |
| DHH | 1.136620315 | 50846 |
| BTN1A1 | 1.321681584 | 696 |
| SIRPG | 1.306803216 | 55423 |
| LCE1E | -2.340465305 | 353135 |
| LRRC37A11P | -1.140078565 | 342666 |
| AL603840.1 | -1.77063856 | NA |
| AC000067.1 | 1.330666536 | NA |
| TMX2-CTNND1 | -1.051745178 | 100528016 |
| NOX4 | 1.059352039 | 50507 |
| SERTAD4 | 1.038614423 | 56256 |
| ABCA4 | -3.087527318 | 24 |
| AC109446.3 | 1.233467783 | NA |
| AC005089.1 | 1.199629956 | NA |
| AC025884.1 | 2.233017459 | NA |
| PPY | 1.68372355 | 5539 |
| NKAIN3-IT1 | -1.078255935 | 643763 |
| AC087286.2 | -1.235784108 | NA |
| AC126773.4 | 1.151897967 | NA |
| C6 | -3.417117172 | 729 |
| CAPN6 | 1.655026894 | 827 |
| BX284613.2 | -1.863836014 | NA |
| HNRNPA1P27 | -1.047397384 | 664721 |
| AL161785.2 | -1.358843941 | NA |
| AL157396.1 | -1.800784885 | NA |
| IGKV3-15 | 2.371856087 | 28913 |
| RN7SKP83 | -2.249755092 | 106479131 |
| CST7 | 1.018679027 | 8530 |
| TRBV7-9 | 1.547916645 | 28589 |
| COL9A3 | 1.18183223 | 1299 |
| CHL1-AS2 | -1.322354167 | 101927174 |
| AC025176.1 | 1.413178435 | NA |
| AL450332.1 | -1.53960992 | NA |
| MIXL1 | 1.015828101 | 83881 |
| AC016831.6 | -1.246774817 | NA |
| FOXG1-AS1 | -1.044632269 | 103695363 |
| AP000864.1 | 1.177049637 | NA |
| AL021918.1 | -1.311618621 | NA |
| ABHD17AP5 | 1.032246954 | 91219 |
| MKI67 | 1.208834076 | 4288 |
| MTCO3P12 | -3.047818421 | 107075270 |
| NCMAP | -1.791290461 | 400746 |
| AC137056.1 | -2.222060611 | NA |
| HOXA13 | 2.032526685 | 3209 |
| AL033528.1 | 1.043659275 | NA |
| HIST1H1E | 1.084912329 | NA |
| AC112656.1 | 1.154779551 | NA |
| LINC00958 | 1.339590383 | 100506305 |
| EXPH5 | -1.234322917 | 23086 |
| LINC01088 | -1.779888838 | 100505875 |
| OR51E1 | 1.575403087 | 143503 |
| OTOGL | -1.11347673 | 283310 |
| OIT3 | 1.126781319 | 170392 |
| WDR49 | -1.218741204 | 151790 |
| KANK4 | 1.139604896 | 163782 |
| AL359644.1 | -1.404582154 | NA |
| ST7-AS2 | -2.073869247 | 93654 |
| PON1 | -1.405206661 | 5444 |
| AC026780.2 | -1.346480549 | NA |
| CYP4F22 | 1.380932618 | 126410 |
| LINC02030 | -2.570389507 | 105377098 |
| AL590762.2 | 1.236787399 | NA |
| AC004947.2 | -1.235448627 | NA |
| SLC13A4 | -2.447824218 | 26266 |
| AC106738.1 | 1.851293234 | NA |
| FAM107A | -1.025860338 | 11170 |
| ZNF849P | 1.195682087 | 100130108 |
| VWA3B | -1.297818964 | 200403 |
| AC067930.4 | 1.001572561 | NA |
| TMPRSS9 | 1.244086314 | 360200 |
| GAL | 1.338549206 | 51083 |
| AC020658.4 | 1.476859316 | NA |
| HLA-DQB2 | 1.471849605 | 3120 |
| TEX15 | 1.0027301 | 56154 |
| TSSC2 | 1.079019354 | 650368 |
| TYR | 2.031810605 | 7299 |
| AC018861.2 | -1.87271234 | NA |
| RASL12 | -1.20204313 | 51285 |
| HIST1H2BL | 1.161262032 | NA |
| TSPAN18 | 1.099001517 | 90139 |
| AC010524.1 | 1.300282229 | NA |
| LINC01726 | -1.984807567 | 101929608 |
| AP001107.3 | 1.339786005 | NA |
| LINC01711 | 1.285640978 | 79160 |
| DRAXIN | 1.046097772 | 374946 |
| WNT7B | 1.105433875 | 7477 |
| LINC02200 | -1.321434028 | 102467214 |
| ARHGAP40 | -1.660500589 | 343578 |
| PRSS50 | -1.168958297 | 29122 |
| LINC01978 | 1.019658218 | 101928738 |
| HNRNPCP8 | 1.107962694 | 100131842 |
| HVCN1 | -1.048858708 | 84329 |
| AC108463.1 | 1.256598289 | NA |
| AC140912.1 | -1.10209622 | NA |
| TNR | -1.250402219 | 7143 |
| GRAMD1C | -1.045999126 | 54762 |
| LINC02232 | -1.287602491 | 401134 |
| AL713899.1 | 1.042514965 | NA |
| SEL1L2 | 1.114209961 | 80343 |
| FOXN4 | 1.065538153 | 121643 |
| LINC02538 | 1.129483399 | 401286 |
| SEZ6L | -1.218745064 | 23544 |
| FOXM1 | 1.105852567 | 2305 |
| AC090877.2 | 1.344402581 | NA |
| DBET | 1.790458758 | 100419743 |
| FBN3 | 1.210640505 | 84467 |
| CPAMD8 | -2.351596131 | 27151 |
| C9orf129 | -1.188259955 | 445577 |
| COL12A1 | 1.063510246 | 1303 |
| ANGPT4 | -1.247028856 | 51378 |
| SLCO1C1 | -1.011745462 | 53919 |
| AC006946.2 | 1.10341889 | NA |
| SLC2A12 | -1.028806459 | 154091 |
| OBP2A | 1.472512339 | 29991 |
| RANBP3L | -1.014874354 | 202151 |
| PCDHGA3 | -2.006893505 | 56112 |
| LINC02182 | 1.050513503 | 101928880 |
| RYR1 | -1.038049261 | 6261 |
| AC116609.1 | 1.565923498 | NA |
| AC093520.1 | 1.187573785 | NA |
| LINC02008 | -1.636133975 | 105377180 |
| SRY | -4.590086108 | 6736 |
| ZSCAN10 | -2.129142606 | 84891 |
| TOP2A | 1.254481621 | 7153 |
| PRICKLE2-AS2 | -1.796152578 | 100874242 |
| AC117500.3 | 1.259111487 | NA |
| DCHS2 | -1.225459985 | 54798 |
| LINC01998 | -1.343383035 | 107986046 |
| MAPK4 | -1.011774796 | 5596 |
| LINC02056 | -1.455421433 | 102477328 |
| AC067942.2 | -1.002211286 | NA |
| PROK1 | -1.201943787 | 84432 |
| NAT2 | 1.09594905 | 10 |
| XG | 1.480244218 | 7499 |
| AC104843.1 | 1.096049872 | NA |
| AL049539.1 | 1.104521771 | NA |
| PCDHGA11 | -2.419356733 | 56105 |
| AC122710.1 | -1.792755133 | NA |
| SLIT2 | 1.146819709 | 9353 |
| HLA-DQA1 | 1.282636601 | 3117 |
| THRSP | -1.212940918 | 7069 |
| AACSP1 | 1.147740721 | 729522 |
| AC073636.1 | -1.672988619 | NA |
| HOXB-AS2 | 2.588171056 | 100874350 |
| TTLL6 | -1.459740324 | 284076 |
| MT2P1 | -1.211029617 | 4503 |
| AC002451.1 | -1.120007557 | NA |
| AC010997.5 | 1.247733906 | NA |
| TMC3 | -2.935918139 | 342125 |
| AC084357.3 | 1.726981737 | NA |
| AC011603.1 | 1.286354744 | NA |
| HSPB8 | -1.084470098 | 26353 |
| 7SK | 1.140631363 | NA |
| SMIM32 | 1.32618561 | 389332 |
| MIR657 | 1.058049124 | 724027 |
| SLC15A2 | -1.010376009 | 6565 |
| FGF10 | 1.577374815 | 2255 |
| CYP4F60P | 1.163095994 | 107126294 |
| MYOC | -3.288912337 | 4653 |
| S100A4 | 1.106732283 | 6275 |
| AC104248.1 | -2.001834893 | NA |
| MCRIP2P1 | 1.097060097 | 102388891 |
| PRSS56 | 1.500727158 | 646960 |
| CSDC2 | -1.271228261 | 27254 |
| ENOX1-AS2 | -1.491882929 | 100874130 |
| AC091182.1 | 1.381858801 | NA |
| GMNC | -3.712509706 | 647309 |
| CHL1 | -1.158655116 | 10752 |
| AC004022.1 | -2.208516717 | NA |
| FO393415.1 | -1.120977366 | NA |
| LINC01094 | -1.054550087 | 100505702 |
| HIST1H2BO | 1.079540515 | NA |
| AC106785.2 | 2.174765474 | NA |
| LRRC37A7P | -1.073046148 | 100421589 |
| AL161804.1 | -1.468970977 | NA |
| SNORD3B-2 | 1.397227229 | 780852 |
| AL158058.1 | -2.279718184 | NA |
| LINC01354 | -1.087256486 | 100506795 |
| SNORA73B | 1.133147121 | 26768 |
| LINC02718 | 1.018978684 | 105376592 |
| ACKR4 | -1.346992835 | 51554 |
| AC090625.2 | -1.115981479 | NA |
| ELOVL2-AS1 | -1.284832821 | 100506409 |
| MIR548AN | -1.684812245 | 100616144 |
| AL358154.1 | -1.878755159 | NA |
| AC016831.3 | -1.749333006 | NA |
| LRRC37A6P | -1.145104548 | 387646 |
| LRTM1 | 1.365603014 | 57408 |
| TXNDC2 | -1.028845791 | 84203 |
| AC136475.1 | -1.01412332 | NA |
| FOXE1 | 1.694842422 | 2304 |
| AC010307.2 | 1.37268182 | NA |
| LINC01388 | -1.007136165 | 101929179 |
| AC108156.1 | -1.210124241 | NA |
| VN1R51P | 1.022254951 | 100312801 |
| RPLP0P2 | 1.075768814 | 113157 |
| GBA3 | -2.547763474 | 57733 |
| LMO7DN-IT1 | 1.658567174 | 104326189 |
| AGXT2 | 1.878966269 | 64902 |
| IQGAP3 | 1.048599147 | 128239 |
| RPL31P11 | 1.080270072 | 641311 |
| FAM167A-AS1 | -1.33326788 | 83656 |
| IQCA1L | -1.577621728 | 392843 |
| AC002480.2 | -1.368597987 | NA |
| CNGA3 | -1.805460509 | 1261 |
| HSPB2-C11orf52 | -1.785623227 | 100528019 |
| MYOM1 | -1.031958792 | 8736 |
| LINC01338 | -1.398087448 | 102546175 |
| AC124303.1 | -1.319989001 | NA |
| AC107398.3 | -1.113019149 | NA |
| AP000697.1 | 1.397691785 | NA |
| LINC01994 | -2.163673133 | 401103 |
| MTTP | -1.259260255 | 4547 |
| AL391845.2 | -1.658908288 | NA |
| AC004816.2 | 1.099810182 | NA |
| FNDC1 | 1.400136822 | 84624 |
| TEX19 | 1.285421127 | 400629 |
| AC114316.1 | -1.401786878 | NA |
| SLC28A3 | -1.431650709 | 64078 |
| LINC01447 | 1.776877653 | 101929086 |
| LRRC8E | 1.101099665 | 80131 |
| AC124856.1 | 1.042416027 | NA |
| MED28P3 | -1.115137622 | 100131051 |
| TAT | -1.202988814 | 6898 |
| IGHV3-49 | 2.438997687 | 28423 |
| AC097713.1 | 1.659378195 | NA |
| LINC01546 | -1.684687332 | 100129464 |
| SIDT1 | -1.116999927 | 54847 |
| AC026316.2 | -1.441963589 | NA |
| CAPSL | -3.082763472 | 133690 |
| NKX2-8 | 1.175240888 | 26257 |
| MT-TS2 | 1.028702659 | NA |
| MUC12 | 1.053547532 | 10071 |
| DRD3 | -2.552584316 | 1814 |
| TGM5 | -1.867532551 | 9333 |
| PTGDR | 1.251323894 | 5729 |
| ARHGAP27P2 | 1.055255825 | 440461 |
| ICOS | 1.479360304 | 29851 |
| INHBA-AS1 | -1.575508249 | 285954 |
| AC092162.2 | -1.273626564 | NA |
| GOLGA8UP | 1.094538253 | 100507067 |
| PTX3 | 1.130061801 | 5806 |
| NXPE2 | -1.062708408 | 120406 |
| AC016573.1 | -1.438083727 | NA |
| GDI2P1 | -2.303682006 | 2667 |
| MTND6P4 | -1.63059307 | 106478943 |
| AC116348.1 | 1.448813339 | NA |
| TGFBI | 1.067849878 | 7045 |
| AL137025.1 | -1.075429967 | NA |
| CFAP221 | -1.10309882 | 200373 |
| CD38 | -1.085709092 | 952 |
| NXPH1 | -1.123953496 | 30010 |
| AL356961.1 | 1.062965744 | NA |
| RDH10-AS1 | 1.097085676 | 101926926 |
| LINC02683 | -1.321925782 | 101928132 |
| LINC02518 | -2.060792383 | 105377957 |
| SIX6 | 2.206279027 | 4990 |
| SMIM35 | -1.697831336 | 100526771 |
| HSPA7 | 1.283525316 | 3311 |
| AC114291.1 | 1.277507971 | NA |
| AC131571.1 | -1.250089377 | NA |
| NEIL3 | 1.128470255 | 55247 |
| ACBD7 | -1.295854576 | 414149 |
| RNA5SP39 | -1.909992506 | 100873371 |
| DCDC2 | -1.459569825 | 51473 |
| AC104574.1 | -2.486082687 | NA |
| FOLR3 | 1.208484683 | 2352 |
| AC084880.1 | -1.144685574 | NA |
| AL121904.1 | 1.933963676 | NA |
| TDGF1 | -1.058216872 | 6997 |
| CCL14 | 1.282589768 | 6358 |
| IGLV5-45 | 2.116521296 | 28781 |
| HCG4 | -1.250829214 | 54435 |
| PCARE | -1.192677331 | 388939 |
| CRNDE | 1.219147579 | 643911 |
| AC006971.1 | -1.069840194 | NA |
| LINC01982 | -2.221855414 | 105371830 |
| LINC01055 | -3.345566677 | 103752581 |
| ABHD17AP4 | 1.02841708 | 729495 |
| RPL21P108 | -1.167838902 | 100271434 |
| LINC02343 | -2.050026268 | 105370158 |
| FER1L6-AS2 | 1.19374968 | 157376 |
| AC091891.1 | 1.267592414 | NA |
| AL590428.1 | -1.254769461 | NA |
| KSR2 | -1.379805312 | 283455 |
| OLFM4 | -1.620705166 | 10562 |
| AC073130.3 | 1.003975081 | NA |
| AL355596.1 | -2.2953899 | NA |
| LINC02156 | -2.019825137 | 111082992 |
| LINC01943 | 1.266726058 | 101928173 |
| IGSF1 | -1.505547495 | 3547 |
| LINC02814 | -1.674196295 | 112267873 |
| CYP4F35P | 1.188415413 | 284233 |
| AL353753.1 | -1.985703471 | NA |
| ARHGAP36 | -1.576019442 | 158763 |
| GPR151 | -1.154699954 | 134391 |
| HPD | -2.236678335 | 3242 |
| TTPA | -1.153617519 | 7274 |
| IGFBP2 | 1.314081543 | 3485 |
| GSDMA | 1.171459794 | 284110 |
| AC099794.1 | 1.002444346 | NA |
| AC084880.4 | -2.228811225 | NA |
| PPIAP39 | -1.515111277 | 111082963 |
| EFHC2 | -1.56562336 | 80258 |
| DLX1 | 1.222665443 | 1745 |
| AC092652.1 | -1.615418228 | NA |
| ADGRF5P1 | -1.01865498 | 389740 |
| AP003472.1 | -1.196498526 | NA |
| MYMX | 1.115735015 | 101929726 |
| BHLHA15 | -1.468105449 | 168620 |
| SLC22A8 | -2.969594775 | 9376 |
| SELL | -1.780225269 | 6402 |
| PITX3 | 1.062461522 | 5309 |
| WNT16 | -2.386423418 | 51384 |
| SECTM1 | 1.002029971 | 6398 |
| MT1X | -1.149743235 | 4501 |
| GRM5 | -1.769715945 | 2915 |
| RD3 | -3.396109257 | 343035 |
| SFTPA2 | -1.139334997 | 729238 |
| AL138902.1 | -1.473788259 | NA |
| LINC00927 | -1.708648148 | 283688 |
| LRRC77P | -2.250450815 | 646168 |
| OBI1-AS1 | -1.275133951 | 100874222 |
| OSTN | -2.125543147 | 344901 |
| AC112206.2 | -1.370743549 | NA |
| NCR3LG1 | -1.15237367 | 374383 |
| AC092957.1 | -2.090390541 | NA |
| CXCR3 | 1.31871141 | 2833 |
| AC098818.1 | -1.331487762 | NA |
| PIPOX | -1.119635845 | 51268 |
| ADH1B | -1.292446968 | 125 |
| SFTPA1 | -2.337054076 | 653509 |
| FOXR2 | 2.429127023 | 139628 |
| LINC00973 | 1.097389931 | 100506377 |
| AC027306.1 | -1.648777558 | NA |
| AL162725.2 | -1.755903994 | NA |
| MINAR2 | 1.176795358 | 100127206 |
| IL2RB | 1.094650426 | 3560 |
| WNT9B | -1.183234455 | 7484 |
| XCL1 | 1.15619747 | 6375 |
| LINC00499 | -2.310373793 | 100874047 |
| LINC02690 | -1.804241943 | 105376658 |
| LIPF | -5.698770428 | 8513 |
| MTCO2P12 | -1.745434923 | 107075310 |
| MTATP8P1 | -1.672726177 | 106480795 |
| C1orf189 | -2.702828186 | 388701 |
| AL603832.3 | 1.079155421 | NA |
| KBTBD12 | -1.128102954 | 166348 |
| HP | -2.989360723 | 3240 |
| HKDC1 | -1.188518612 | 80201 |
| AC027288.3 | -1.88329389 | NA |
| AL035420.1 | -1.263912655 | NA |
| TCAM1P | 1.166620802 | 146771 |
| AGMO | -1.234022879 | 392636 |
| P2RY1 | -1.172046958 | 5028 |
| PAPPA | -1.249434615 | 5069 |
| HOXA10 | 1.884386031 | 3206 |
| AL357093.2 | -2.794297054 | NA |
| AC104009.1 | 1.149520367 | NA |
| LINC02067 | -1.465651612 | 101243545 |
| AL355073.1 | -1.100261293 | NA |
| GRIN3A | -1.568297269 | 116443 |
| LINC01901 | 1.25345954 | 105372080 |
| BSPRY | -1.107218718 | 54836 |
| FSTL4 | 1.135067805 | 23105 |
| MTND1P23 | 1.634312645 | 100887749 |
| GOLT1A | -2.124304423 | 127845 |
| TPSB2 | 1.431945455 | 64499 |
| PPP1R2P1 | -1.072209387 | 100507444 |
| AL008638.6 | -1.52621583 | NA |
| LINC01215 | 1.089066361 | 101929623 |
| BHMT | -1.404816361 | 635 |
| LINC01564 | -1.532150174 | 101927171 |
| AL009178.2 | 1.384860049 | NA |
| AC003973.2 | 1.268854745 | NA |
| DLX6 | 1.183394289 | 1750 |
| AC022498.1 | -1.933297475 | NA |
| FOXD3 | 1.464467278 | 27022 |
| AF131216.3 | -1.105009179 | NA |
| HSD17B2 | 1.408692143 | 3294 |
| PCDHB8 | -1.393689182 | 56128 |
| SNX31 | -1.614925027 | 169166 |
| CCL23 | 1.395293051 | 6368 |
| ANKRD20A11P | 1.192099251 | 391267 |
| CDC14C | -1.553424294 | 168448 |
| AC004264.2 | -1.022537454 | NA |
| AC092162.3 | -1.593745437 | NA |
| LINC01840 | -1.294705034 | 100874079 |
| AL133371.3 | -1.307969289 | NA |
| KLK3 | 2.391196814 | 354 |
| CD3G | 1.095831924 | 917 |
| OR2M4 | 1.382574703 | 26245 |
| ZNF603P | -1.126820147 | 493821 |
| HOXA10-AS | 2.359705751 | 100874323 |
| SAA2 | -3.24624332 | 6289 |
| AC015656.1 | 1.195632145 | NA |
| AL049830.3 | 1.102533044 | NA |
| AL157938.1 | -1.006129229 | NA |
| PAPLN | -1.01794808 | 89932 |
| KLHDC7A | -3.861558099 | 127707 |
| AC124312.2 | -1.008394799 | NA |
| TSPAN19 | 1.171937241 | 144448 |
| AC141557.2 | 1.135196548 | NA |
| AC068643.1 | -1.062113686 | NA |
| C12orf40 | -1.687739645 | 283461 |
| MAP1LC3C | -2.164895223 | 440738 |
| KCNJ13 | -2.748771952 | 3769 |
| ADAMTS14 | 1.026737755 | 140766 |
| AC116348.2 | 1.707207679 | NA |
| ALX3 | -1.269956109 | 257 |
| AC122710.2 | 1.006891904 | NA |
| NDST4 | -1.809796382 | 64579 |
| AC103563.2 | 1.235754331 | NA |
| MT1M | -1.316990578 | 4499 |
| LINC02675 | -1.35046035 | 112267862 |
| LMNTD1 | -1.39552324 | 160492 |
| AC084864.1 | -1.868734499 | NA |
| DDX11L16 | 1.985624511 | 727856 |
| AC139491.2 | -1.276194298 | NA |
| SELENOKP3 | -1.264160038 | 100287632 |
| AC092040.1 | -1.091880303 | NA |
| TCERG1L | 1.22695976 | 256536 |
| HOXD12 | 1.961355017 | 3238 |
| PLCXD2 | -1.097204726 | 257068 |
| AC025884.2 | 2.19401289 | NA |
| S100A7 | 1.384019111 | 6278 |
| IAPP | 1.200329864 | 3375 |
| ACSM5 | -1.027360597 | 54988 |
| AC078922.1 | -1.81355536 | NA |
| AC006272.1 | 1.079296952 | NA |
| BUB1B | 1.121743011 | 701 |
| AL133371.1 | -1.624932719 | NA |
| NPY4R2 | 1.835835452 | 100996758 |
| AC016717.2 | -1.379897046 | NA |
| SSTR1 | -1.43921157 | 6751 |
| LINC00407 | -1.072495975 | 106144541 |
| AC093809.1 | -1.522864152 | NA |
| LINC00543 | 1.379050175 | 100132234 |
| TMPRSS7 | -2.391431249 | 344805 |
| COLCA1 | -1.408954083 | 399948 |
| C5orf64-AS1 | -1.336948205 | 100506526 |
| SCNN1B | -2.291284873 | 6338 |
| CHL1-AS1 | -1.323501419 | 101927193 |
| CD2 | 1.172936105 | 914 |
| RPL12P7 | -1.022237526 | 326275 |
| AC005162.3 | -1.161863182 | NA |
| ZBTB16 | -1.023148314 | 7704 |
| ADGRF5P2 | -1.162153244 | 441416 |
| SLC1A2 | -1.00305043 | 6506 |
| LYVE1 | -1.395506937 | 10894 |
| AL161733.1 | 1.328507659 | NA |
| FRMPD2B | -1.840694251 | 728798 |
| NR1H4 | -1.600833435 | 9971 |
| AC011270.2 | 1.469152165 | NA |
| PLA1A | -1.006919589 | 51365 |
| LINC02058 | -1.412406311 | 107984117 |
| AL451042.2 | 1.021424788 | NA |
| SLC15A5 | -1.080129067 | 729025 |
| KCNA5 | -1.308405202 | 3741 |
| AP002530.1 | -1.229585159 | NA |
| PRLHR | -1.94894772 | 2834 |
| AC008554.1 | 1.143137125 | NA |
| GRIA1 | -1.044619028 | 2890 |
| IGKV3D-20 | 2.212659876 | 28874 |
| AL133279.3 | -1.783722038 | NA |
| VEPH1 | -1.700443377 | 79674 |
| ARSF | -1.857951566 | 416 |
| RHEX | 1.232379205 | 440712 |
| AC016910.1 | -1.131452279 | NA |
| BNIP3P37 | 1.136091855 | 106480285 |
| DYNLRB2 | -1.074221876 | 83657 |
| CMYA5 | -1.487271632 | 202333 |
| AL049541.1 | -1.937210979 | NA |
| TLX2 | 1.249387707 | 3196 |
| PAPPA-AS2 | -1.062761508 | 103611155 |
| HS3ST3A1 | 1.204313237 | 9955 |
| ACOT6 | -1.298826444 | 641372 |
| LTF | -3.25601303 | 4057 |
| CNTN6 | -1.336165802 | 27255 |
| RPSAP33 | 1.025312846 | 647158 |
| AP000763.2 | -1.689291001 | NA |
| ADH1C | 1.098221651 | 126 |
| AL121757.1 | -1.723318529 | NA |
| DAAM2 | -1.004153355 | 23500 |
| CYYR1-AS1 | -1.083966234 | 100996571 |
| GOLGA2P9 | 1.504089177 | 440518 |
| AC068254.2 | 1.072107079 | NA |
| FBXO17 | -1.316408273 | 115290 |
| ADAMDEC1 | 1.547883247 | 27299 |
| PLIN1 | -1.279031049 | 5346 |
| HMGA1P7 | -1.518544551 | 387065 |
| HECW2-AS1 | 1.205819103 | 101927482 |
| FAR2P2 | -1.325724949 | 100216479 |
| CNTNAP5 | -1.308976945 | 129684 |
| LINC01234 | 1.018970922 | 100506465 |
| LINC01831 | -1.577167171 | 101927331 |
| CLEC9A | -1.236562144 | 283420 |
| CBX1P2 | -1.037083906 | 100147811 |
| AC107057.1 | 2.181966485 | NA |
| P2RY12 | -1.03328031 | 64805 |
| AC008127.1 | -1.192175167 | NA |
| THBS4 | -1.154624892 | 7060 |
| LINC00475 | -2.922839302 | 158314 |
| GABRG1 | -1.335463367 | 2565 |
| AC026992.1 | -1.163557327 | NA |
| VGLL2 | 1.808677629 | 245806 |
| IGLV5-37 | 1.460031888 | 28783 |
| PLA2G5 | -1.754355214 | 5322 |
| KCNG4 | -1.517604979 | 93107 |
| AC079140.4 | -1.159410469 | NA |
| Z82196.1 | -1.903865768 | NA |
| AC005520.3 | -1.046600942 | NA |
| IL20RA | -1.531200815 | 53832 |
| SUCLA2P1 | -1.217600008 | 646520 |
| AC113349.1 | -2.410520463 | NA |
| RAMP3 | -1.235387961 | 10268 |
| AC117394.2 | -1.122913475 | NA |
| AC092809.2 | -1.059504306 | NA |
| TRAF6P1 | -1.403889731 | 644871 |
| EXOC5P1 | 1.078804179 | 644548 |
| AL390755.1 | -1.285912295 | NA |
| AC007663.2 | 1.038315372 | NA |
| AC004147.3 | -2.061195359 | NA |
| PRLR | -2.001090888 | 5618 |
| SAA1 | -4.211249858 | 6288 |
| CHRND | 1.078934908 | 1144 |
| AC008060.1 | 1.022912284 | NA |
| PCDHGA7 | -1.625335553 | 56108 |
| DUXAP8 | 1.005336222 | 503637 |
| COL11A1 | 1.029283708 | 1301 |
| NAA11 | -1.453827013 | 84779 |
| AC022710.1 | -1.219756225 | NA |
| LINC01749 | 1.261831547 | 63930 |
| HIST1H2AL | 1.305669718 | NA |
| AL355512.1 | 1.213998318 | NA |
| DMKN | -1.219770023 | 93099 |
| NT5C1A | -1.130701321 | 84618 |
| SCGN | -2.342221523 | 10590 |
| IGF2BP3 | 1.446239751 | 10643 |
| LINC00571 | -1.003333175 | 100874188 |
| AC092042.2 | -1.295981832 | NA |
| AC005381.1 | 1.48041014 | NA |
| GJA3 | 1.413845517 | 2700 |
| APOB | -1.547008408 | 338 |
| EVX1-AS | 1.511977678 | 101410536 |
| AC068790.1 | -1.317774514 | NA |
| TNRC18P2 | -1.353151145 | 27320 |
| INSRR | 1.006514687 | 3645 |
| LINC01933 | -1.039774225 | 101927115 |
| AP000438.1 | -1.057392079 | NA |
| AC019118.2 | -1.477158978 | NA |
| SLED1 | 1.060442317 | 643036 |
| LINC02634 | -1.366169076 | 105376493 |
| AC005336.1 | -1.182662395 | NA |
| AC063952.3 | -3.984873818 | NA |
| AL590705.2 | 1.118421714 | NA |
| F5 | -1.85459755 | 2153 |
| TMEM151B | -1.139102821 | 441151 |
| AC006148.1 | -1.05787839 | NA |
| GSX1 | 1.119627695 | 219409 |
| AC073324.1 | -3.039647173 | NA |
| IL6 | -1.990980622 | 3569 |
| AC099681.1 | -1.470184706 | NA |
| GABRR3 | -1.660455527 | 200959 |
| NPHP3-AS1 | -1.090618445 | 348808 |
| KRTAP16-1 | 1.181299584 | 100505753 |
| AC069281.1 | -1.083276823 | NA |
| CLYBL-AS1 | -2.058099523 | 101927465 |
| CX3CR1 | -1.140149661 | 1524 |
| CRB3P1 | -2.544970942 | 106480279 |
| SCGB3A2 | -3.126303691 | 117156 |
| IQCA1 | -1.187506769 | 79781 |
| IGLC3 | 1.743380477 | 3539 |
| AL589743.5 | 1.15716736 | NA |
| PCDHA4 | -1.595612869 | 56144 |
| AC108868.1 | 1.39121755 | NA |
| PLCH1 | -1.01974671 | 23007 |
| TMPRSS11D | 1.106298049 | 9407 |
| AC004691.2 | -1.525701514 | NA |
| ANKUB1 | -2.025078937 | 389161 |
| CLEC12A | 1.197132256 | 160364 |
| LINC01559 | -1.598380235 | 283422 |
| HOXA9 | 1.962231528 | 3205 |
| NTS | -4.012669084 | 4922 |
| AC092675.2 | -2.471809077 | NA |
| RASSF9 | -2.166840019 | 9182 |
| SNORC | -1.279009311 | 389084 |
| LINC02587 | -3.215119539 | 101927524 |
| MYH11 | -1.514815075 | 4629 |
| AC016877.3 | 1.344686475 | NA |
| LINC00944 | 1.106863895 | 387895 |
| FAM181A-AS1 | -1.332837663 | 283592 |
| AC131009.2 | 1.022444788 | NA |
| IGHG1 | 1.639980663 | 3500 |
| OR2H5P | -1.140722392 | 26713 |
| AC018445.1 | 1.199812277 | NA |
| CSF3 | -2.495930057 | 1440 |
| DIRAS2 | -1.085515045 | 54769 |
| MROH2A | 1.100523456 | 339766 |
| AC004080.1 | 1.541091849 | NA |
| PCDHA3 | -1.2285634 | 56145 |
| FSTL5 | -1.337614489 | 56884 |
| TUBAL3 | -1.685235785 | 79861 |
| AL158840.1 | -1.358927821 | NA |
| TP63 | -1.587422851 | 8626 |
| LINC01248 | 1.086404526 | 102723818 |
| HMGA2-AS1 | 1.247939338 | 100129940 |
| AP003472.2 | -1.464011468 | NA |
| NCKAP5-IT1 | -1.683227301 | 100874346 |
| AC008080.4 | 1.368043033 | NA |
| RTP1 | -1.253255851 | 132112 |
| FDPSP8 | 1.038854463 | 401981 |
| AC004988.1 | 1.290044101 | NA |
| COL4A6 | -1.166669169 | 1288 |
| TTC29 | -1.266604862 | 83894 |
| LINC00552 | 1.0642762 | 100130386 |
| CASC20 | 1.233852067 | 101929244 |
| AL139351.1 | -1.475700276 | NA |
| C6orf15 | -6.091293778 | 29113 |
| AC021678.2 | -3.322115406 | NA |
| TRIM29 | -1.552362996 | 23650 |
| CLDN19 | -4.161092285 | 149461 |
| AC090950.2 | -1.018946838 | NA |
| AL139002.1 | -1.923138235 | NA |
| STEAP2-AS1 | -1.448109683 | 100874100 |
| GATD3A | 1.114662634 | 8209 |
| RPS6KA2-AS1 | -1.202791675 | 100861523 |
| C4BPA | -1.834485628 | 722 |
| LINC02823 | 1.158183728 | 105369897 |
| LINC01193 | -1.883208137 | 348120 |
| MT1E | -1.014679597 | 4493 |
| NTF4 | 1.332212919 | 4909 |
| AC125611.1 | 1.07575792 | NA |
| AGR3 | -3.818314776 | 155465 |
| MRGPRE | 1.234744 | 116534 |
| RPL22P12 | 1.097300664 | 727747 |
| AL391427.1 | 1.102835964 | NA |
| AC027281.2 | -1.141717566 | NA |
| CALML3-AS1 | -1.490619779 | 100132159 |
| AC068305.2 | -1.151105611 | NA |
| AC006466.1 | -1.093270279 | NA |
| AL359538.1 | 1.047057598 | NA |
| CHRM2 | 1.028075788 | 1129 |
| AF186996.3 | -1.572754768 | NA |
| CALB1 | -1.475570373 | 793 |
| FCGR2C | 1.117680409 | 9103 |
| ITGB4 | -1.071865429 | 3691 |
| AL512631.1 | -1.030142621 | NA |
| AC055720.1 | -1.676537715 | NA |
| C1orf87 | -1.954260571 | 127795 |
| MTCO1P42 | -1.130032144 | 107075283 |
| PIH1D3 | -3.065849408 | 139212 |
| ZNF804B | -1.254020444 | 219578 |
| ETNPPL | -1.195844083 | 64850 |
| TACSTD2 | 1.091260304 | 4070 |
| AF186996.2 | -1.381443871 | NA |
| EPS8L3 | -1.575811327 | 79574 |
| C1orf143 | -2.133107326 | 440714 |
| AC013553.3 | -1.066868621 | NA |
| RPL7AP28 | -2.185529094 | 100271209 |
| LINC01340 | -1.25828649 | 102546227 |
| FEV | 1.368692876 | 54738 |
| LRRC55 | -1.071309138 | 219527 |
| GBP1P1 | -1.261842473 | 400759 |
| AC008619.1 | -1.349227637 | NA |
| AC005832.2 | -1.501794985 | NA |
| AL359757.1 | -1.439040348 | NA |
| DNAH5 | -1.053738732 | 1767 |
| AC063926.1 | 1.189548948 | NA |
| HOXB2 | 1.2427745 | 3212 |
| LGI1 | -1.166410764 | 9211 |
| LRRC31 | -1.249876888 | 79782 |
| MOGAT3 | 1.053022686 | 346606 |
| AP003032.1 | -3.085569499 | NA |
| TRBC1 | 1.271209962 | 28639 |
| PCDHB17P | -1.341354899 | 54661 |
| RNASE3 | -1.238606485 | 6037 |
| SLC26A7 | -1.204741865 | 115111 |
| IGHA1 | 1.595527334 | 3493 |
| RNU1-65P | -1.514340357 | 106481614 |
| KLF2P2 | -1.464025569 | 106480678 |
| OR2W6P | 1.081405942 | 81406 |
| MAB21L2 | -2.559709434 | 10586 |
| AC073941.2 | -1.311073377 | NA |
| C10orf105 | -1.741806719 | 414152 |
| AC026469.1 | 1.004166166 | NA |
| LINC01727 | -1.396243598 | 101929625 |
| AC067773.1 | -1.221870224 | NA |
| NTSR2 | -1.32165361 | 23620 |
| AL161631.1 | -1.516446898 | NA |
| UBASH3A | 1.082149341 | 53347 |
| GRP | -2.292531333 | 2922 |
| LINC00266-1 | -1.50529249 | 140849 |
| NOX3 | -1.585017671 | 50508 |
| AC108718.1 | -1.729143124 | NA |
| OR2A20P | -2.053751793 | 401428 |
| LPL | -1.012726751 | 4023 |
| AL162419.1 | -1.357393549 | NA |
| MTATP8P2 | -1.627066077 | 106479042 |
| NMNAT3 | -1.050224169 | 349565 |
| CCDC33 | -2.390032772 | 80125 |
| AL513318.2 | -1.226537867 | NA |
| RCC2P8 | -1.249751196 | 100129714 |
| AL109809.4 | -1.225127265 | NA |
| G6PC2 | -1.831047555 | 57818 |
| AC007614.1 | -1.409196856 | NA |
| SIX3-AS1 | 1.450996553 | 100506108 |
| AC244517.6 | -1.102452068 | NA |
| NLRP4 | -1.677748253 | 147945 |
| AC061975.5 | -1.357432238 | NA |
| DNAI1 | -1.056924969 | 27019 |
| SLC22A3 | -1.077925631 | 6581 |
| SLC1A7 | -1.130239918 | 6512 |
| ACTN1-AS1 | 1.050800804 | 161159 |
| IGKV3-11 | 1.400141992 | 28914 |
| SLC14A1 | -1.629686035 | 6563 |
| RSPH6A | 1.021727036 | 81492 |
| OXTR | -1.277240708 | 5021 |
| GPX3 | -1.285969152 | 2878 |
| AC103808.3 | 1.704566352 | NA |
| PDX1 | 1.823451662 | 3651 |
| AC010970.1 | 1.698139437 | NA |
| SLC13A2 | -4.095650206 | 9058 |
| MTATP6P2 | -1.31452012 | 100287745 |
| HMGCLL1 | -1.044849345 | 54511 |
| AL161716.1 | -2.065998497 | NA |
| MYBPC1 | -1.065619278 | 4604 |
| TC2N | -1.178617153 | 123036 |
| APOA2 | -3.445892486 | 336 |
| AL133240.1 | -2.10116632 | NA |
| TNFRSF11B | -1.402920104 | 4982 |
| GUCY2F | -1.021844648 | 2986 |
| AF241726.1 | -2.548558393 | NA |
| DNASE1L3 | 1.104348444 | 1776 |
| GZMK | 1.142270482 | 3003 |
| PCDHGA5 | -1.583326554 | 56110 |
| KCNH1 | -1.031664236 | 3756 |
| AC090506.1 | -1.724636232 | NA |
| ALK | -1.004971945 | 238 |
| AC008278.2 | 1.046198148 | NA |
| AC021218.1 | -2.245126433 | NA |
| IRX2 | 1.051409106 | 153572 |
| HIST1H4A | 1.260243695 | NA |
| ABCF2P1 | -1.019006188 | 344653 |
| RPL7L1P8 | -1.257352445 | 402152 |
| MTND6P22 | -1.309947878 | 107075261 |
| SLC22A6 | -1.101173472 | 9356 |
| OTX2-AS1 | -4.743851221 | 100309464 |
| TRPA1 | 1.239294538 | 8989 |
| LDHC | 1.567839069 | 3948 |
| IGHV3-43 | 1.983375693 | 28426 |
| PLEKHG4B | 1.088461771 | 153478 |
| AP005264.5 | 1.111822275 | NA |
| AL162726.3 | -1.107769482 | NA |
| AC018761.3 | -1.125608689 | NA |
| AC087379.1 | -1.228971245 | NA |
| DES | -2.086806453 | 1674 |
| AC127381.1 | -2.243035894 | NA |
| BARHL2 | 1.51873941 | 343472 |
| BASP1-AS1 | -1.072793803 | 285696 |
| TMEM171 | 1.097272048 | 134285 |
| LINC02015 | 1.075466219 | 102724550 |
| IL1RL1 | -1.91210912 | 9173 |
| LINC00578 | -1.181404359 | 100505566 |
| FOXD3-AS1 | 1.14047103 | 100996301 |
| AL512785.1 | -1.909815981 | NA |
| RAPSN | -1.333562961 | 5913 |
| CYP2A13 | -2.703897093 | 1553 |
| LINC02470 | -2.522357842 | 100506159 |
| OTX2 | -6.047546815 | 5015 |
| LINC01489 | -1.456569406 | 101928340 |
| TKTL1 | -1.34671326 | 8277 |
| LINC02239 | -1.608836497 | 340107 |
| HPSE2 | -1.414652156 | 60495 |
| AC104793.1 | -1.505944313 | NA |
| LINC01776 | -1.691671398 | 729987 |
| BX322784.1 | -1.041689212 | NA |
| ANKRD55 | -1.23007832 | 79722 |
| FAM205C | -1.197042931 | 100129969 |
| RPH3A | -1.297049509 | 22895 |
| MARVELD3 | -1.151301885 | 91862 |
| EMX1 | 1.112565508 | 2016 |
| AC084346.1 | 1.101815955 | NA |
| LMO7DN | 1.276832317 | 729420 |
| MIR4768 | 1.269436898 | 100616249 |
| RPS8P6 | -2.059170641 | 100270871 |
| AC109309.1 | 1.119797358 | NA |
| AC125603.3 | -1.109371501 | NA |
| AC092652.2 | -1.273815451 | NA |
| AF064860.1 | -1.029163768 | NA |
| PLA2G3 | 1.359002551 | 50487 |
| CCDC198 | -1.879203147 | 55195 |
| LINC01659 | -1.481727074 | 101929374 |
| P2RX1 | -1.088843037 | 5023 |
| GLULP3 | -1.066284501 | 401708 |
| AC079612.1 | -1.791139557 | NA |
| LCE1C | -1.818167522 | 353133 |
| Z98751.2 | -1.235865173 | NA |
| AC016766.1 | -2.216934891 | NA |
