# Supplementary material for: The Recurrent-Specific Regulation Network of Prognostic Stemness-Related Signatures in Low-Grade Glioma
Source: Dis Markers. 2023 Jan 17;2023:2243928. doi: 10.1155/2023/2243928 (PMC9873439; doi:10.1155/2023/2243928)
Supplement: Supplementary Materials — Figure S1: the external validation from Gene Expression Profiling Interactive Analysis (GEPIA): TP53, RB1, CCND1, and CKD4 had a higher expression level in tumor samples than in normal samples in LGG patients. The higher expression level of NCAPG, AURKA, E2F1, TP53, and RB1 was significantly related to worse clinical outcome in patients with LGG. Figure S2: the external validation from Oncomine: NCAPG, AURKA, TP53, RB1, CCND1, and CDK2 had a higher expression level in tumor samples than normal samples in LGG patients. Figure S3: the external validation from UALCAN: (A) the higher expression of NCAPG, AURKA, E2F1, TP53, RB1, CCND1, and CDK4 was related to higher grade in LGG; (B) NCAPG, AURKA, E2F1, CCND1, and CDK4 were related with worse clinical outcome. Figure S4: the external validation from LinkedOmics: the higher expression level of NCAPG, AURKA, E2F1, TP53, RB1, CCND1, and CDK4 was related to worse clinical outcome. Figure S5: the external validation from TISBID: (A) the higher expression level of NCAPG, AURKA, E2F1, CCND1, and CDK4 was related to worse clinical outcome; (B) the higher expression of NCAPG, AURKA, E2F1, TP53, RB1, CCND1, and CDK4 was related to higher grade in LGG. List of differentially expressed genes (DEGs). [file 2243928.f1.zip › Supplementary Materials (1).docx]

**Supplementary Materials**

**
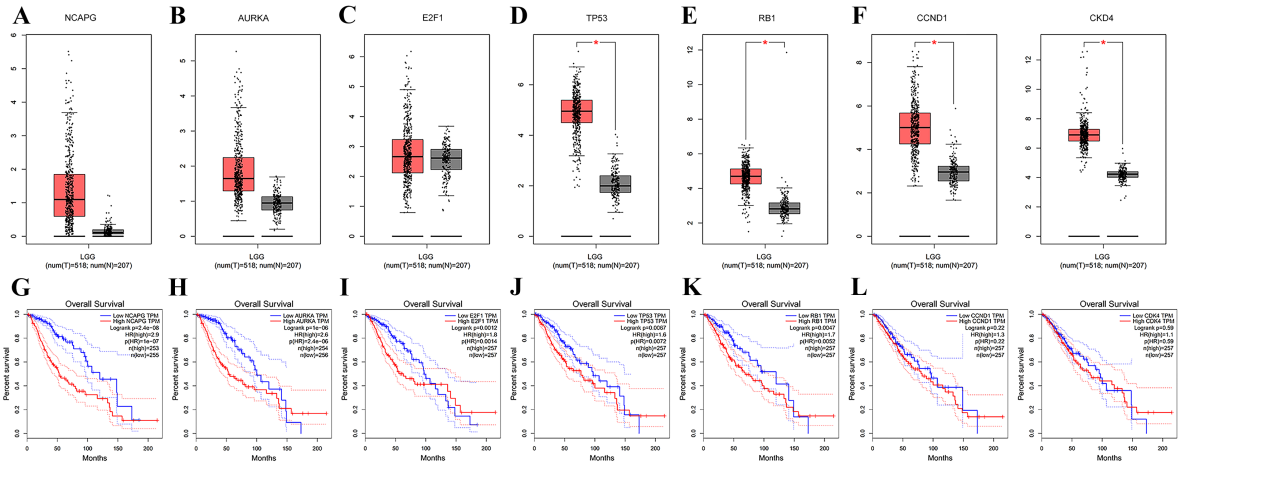
**

Fig. S1 The external validation from Gene Expression Profiling Interactive Analysis (GEPIA): TP53, RB1, CCND1, and CKD4 had a higher expression level in tumor samples than normal samples in LGG patients; The higher expression level of NCAPG, AURKA, E2F1, TP53, and RB1 was significantly related to worse clinical outcome in patients with LGG.


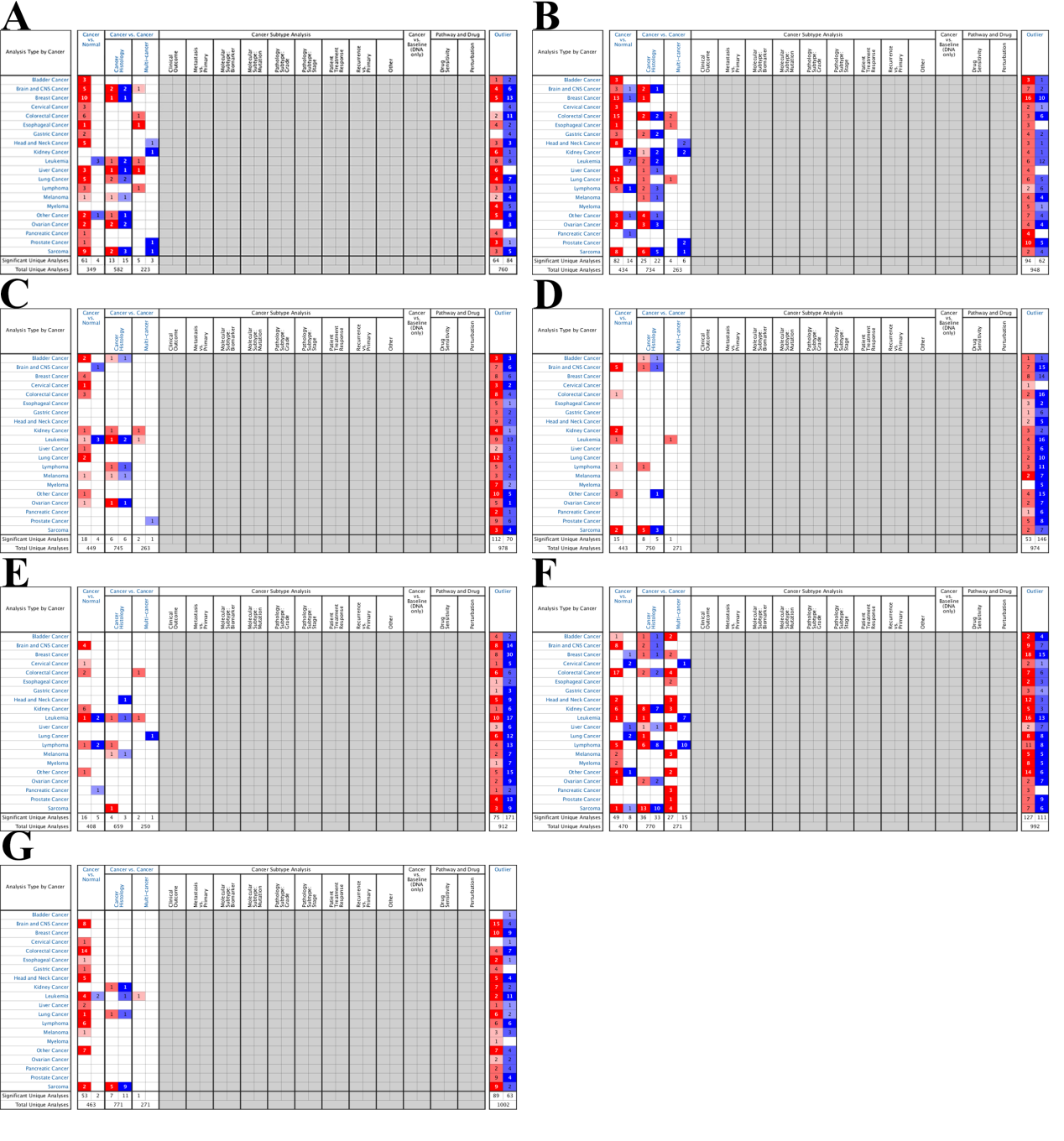


Fig. S2 The external validation from Oncomine: NCAPG, AURKA, TP53, RB1, CCND1, CDK2 had a higher expression level in tumor samples than normal samples in LGG patients;


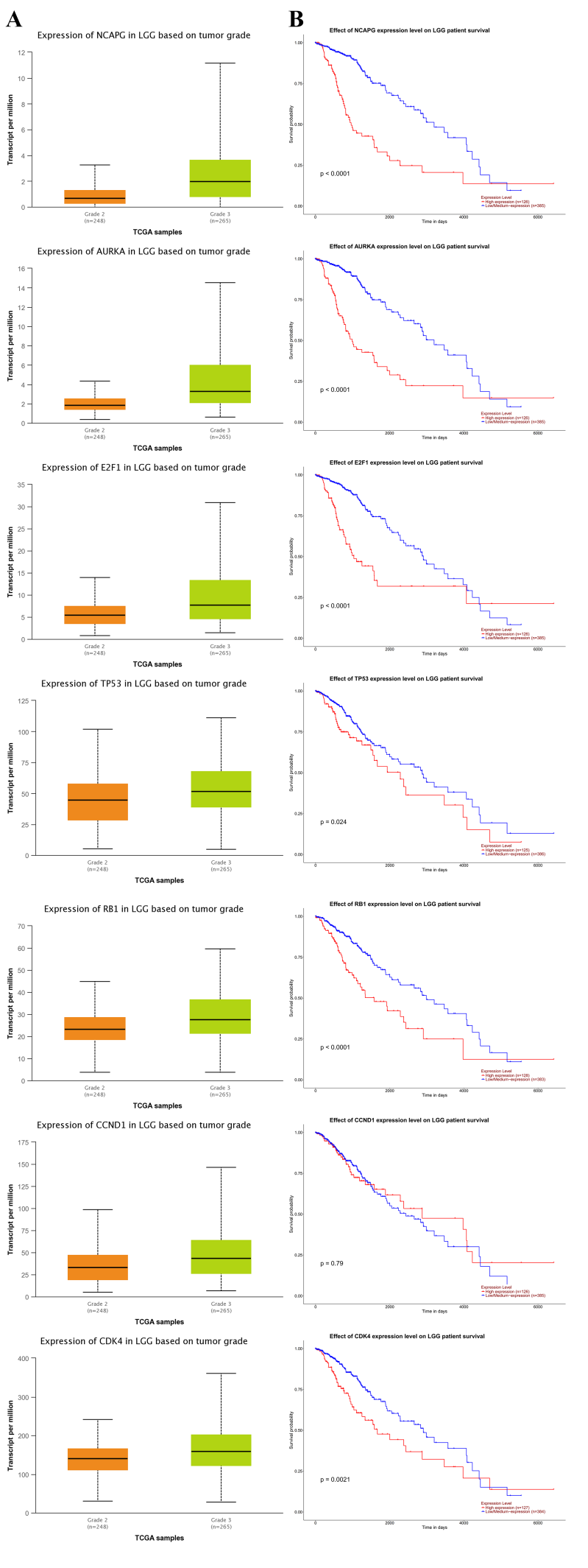


Fig. S3 The external validation from UALCAN: (A) The higher expression of NCAPG, AURKA, E2F1, TP53, RB1, CCND1, and CDK4 were related to higher grade in LGG; (B) NCAPG, AURKA, E2F1, CCND1, and CDK4 related with worse clinical outcome.


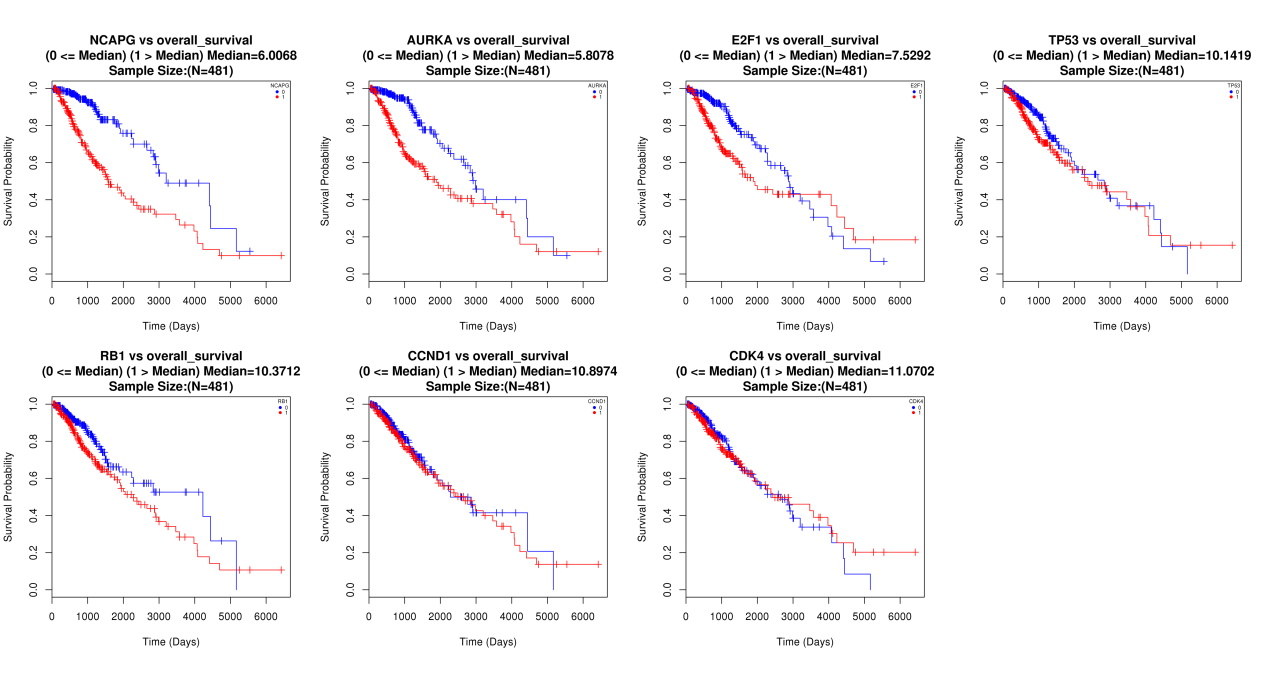


Fig. S4 The external validation from Linkedomics: the higher expression level of NCAPG, AURKA, E2F1, TP53, RB1, CCND1 and CDK4 related to worse clinical outcome.


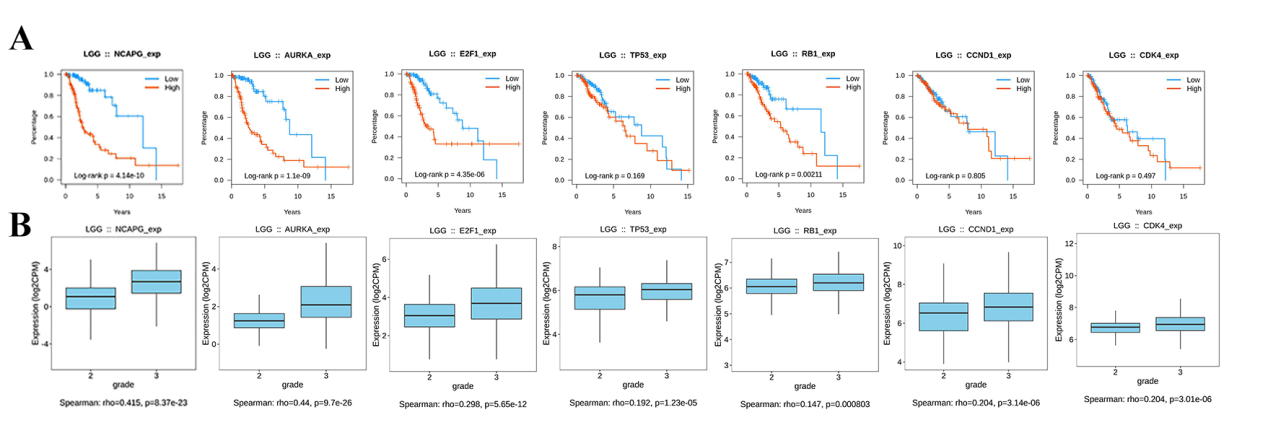


Fig. S5 The external validation from TISBID: (A) the higher expression level of NCAPG, AURKA, E2F1, CCND1 and CDK4 related to worse clinical outcome; (B) The higher expression of NCAPG, AURKA, E2F1, TP53, RB1, CCND1, and CDK4 were related to higher grade in LGG;
